# Per- and Polyfluoroalkyl Substances (PFAS) in PubChem: 7 Million and Growing

**DOI:** 10.1021/acs.est.3c04855

**Published:** 2023-10-23

**Authors:** Emma L. Schymanski, Jian Zhang, Paul A. Thiessen, Parviel Chirsir, Todor Kondic, Evan E. Bolton

**Affiliations:** † Luxembourg Centre for Systems Biomedicine (LCSB), University of Luxembourg, 6 avenue du Swing, 4367 Belvaux, Luxembourg; ‡ National Center for Biotechnology Information, National Library of Medicine, National Institutes of Health, Bethesda, Maryland 20894, United States

**Keywords:** per- and polyfluoroalkyl substances, chemical database, classification, chemical regulation, exposure, high-resolution mass spectrometry, identification, open science

## Abstract

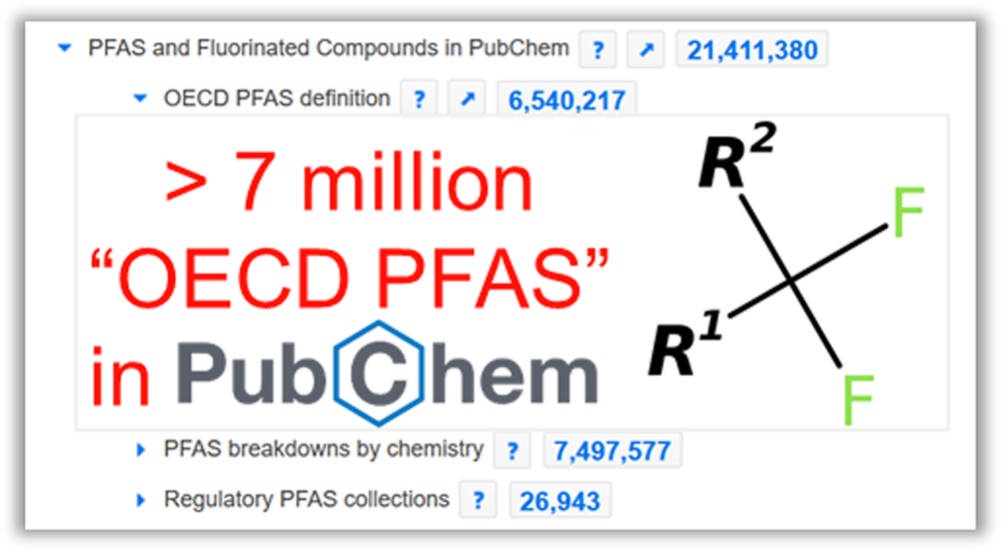

Per- and polyfluoroalkyl substances (PFAS) are of high concern, with calls to regulate them as a class. In 2021, the Organisation for Economic Co-operation and Development (OECD) revised the definition of PFAS to include any chemical containing at least one saturated CF_2_ or CF_3_ moiety. The consequence is that one of the largest open chemical collections, PubChem, with 116 million compounds, now contains over 7 million PFAS under this revised definition. These numbers are several orders of magnitude higher than previously established PFAS lists (typically thousands of entries) and pose an incredible challenge to researchers and computational workflows alike. This article describes a dynamic, openly accessible effort to navigate and explore the >7 million PFAS and >21 million fluorinated compounds (September 2023) in PubChem by establishing the “PFAS and Fluorinated Compounds in PubChem” Classification Browser (or “PubChem PFAS Tree”). A total of 36500 nodes support browsing of the content according to several categories, including classification, structural properties, regulatory status, or presence in existing PFAS suspect lists. Additional annotation and associated data can be used to create subsets (and thus manageable suspect lists or databases) of interest for a wide range of environmental, regulatory, exposomics, and other applications.

## Abstract 2

The open “PFAS and Fluorinated Chemicals in PubChem” collection helps explore millions of PFAS and create relevant subsets for various applications.

## Introduction

Per- and polyfluoroalkyl substances (PFAS) are a group of substances of such high environmental, health, and toxicological concern that there is now a drive to treat PFAS as a class for environmental regulation.^1^ The 2011 definition of PFAS by Buck et al.^2^ included substances as PFAS if they contained two (or more) connected saturated CF_2_ groups. In 2021, the Organisation for Economic Co-operation and Development (OECD) revised the definition of PFAS in ENV/CBC/MONO(2021)25^3^ as follows: “*PFAS are defined as fluorinated substances that contain***at least one fully fluorinated methyl or methylene carbon atom (without any H/Cl/Br/I atom attached to it)***, i.e. with a few noted exceptions, any chemical with at least a perfluorinated methyl group (−CF*_3_*) or a perfluorinated methylene group (−CF*_2_*−) is a PFAS*.”

While early research efforts focused mainly on a very limited list of PFAS, the numbers of documented PFAS are increasing. With the emergence of high-resolution mass spectrometry (HRMS) and the potential for so-called “suspect screening” for contaminants of interest using nontarget analytical techniques,^4,5^ more extensive lists of PFAS became available. The first PFAS list hosted by the NORMAN Suspect List Exchange^6,7^ (hereafter NORMAN-SLE) was the 2015 list contributed by Trier et al.,^8^ which became the basis for the OECD list of ∼4700 PFAS released in 2017.^9,10^ The NORMAN-SLE currently (September 2023) contains 12 PFAS lists.^6,7^ The United States Environmental Protection Agency (US EPA) CompTox Chemistry Dashboard^11^ also hosts chemical lists^12^ and presently (September 2023) hosts 424 lists, including 51 lists matching the PFAS search term,^13,14^ 41 of which contain exclusively fluorinated content. The National Institute of Standards and Technology (NIST) recently coordinated a list (hereafter the “NIST PFAS Suspect List”) of 4948 entries, including expanded homologues and expert contributions.^15^ Several other research efforts have described PFAS lists with various degrees of availability. The OECD PFAS collection of ∼4700 PFAS^9,10^ and the US EPA PFASMASTER list (∼10000 PFAS in 2020, currently 12034 entries in September 2023)^16^ are two of the most frequently used PFAS lists in suspect screening. Both lists also contain entries that are not discrete chemicals, i.e., they also include polymers and substances of Unknown or Variable Composition, Complex Reaction Products, or Biological Materials (UVCBs).^17^ A recent effort with Google and OntoChem investigated the influence of PFAS definition on the number of PFAS extracted from the literature (CORE repository) and patents (Google Patent set), resulting in PFAS lists of between 3457 (CORE, Buck et al.^2^ definition) and 1783651 (Patent set, 2021 OECD PFAS^3^ definition) discrete chemicals.^18^ At the time, over 200000 of these PFAS were not in PubChem,^19,20^ one of the largest open chemistry databases, but were deposited soon thereafter.^18^

There have been several attempts to classify and group PFAS to help answer different questions. The comprehensive OECD efforts^9,10^ contained detailed classifications. The “splitPFAS” method for automated classification was developed and tested on five of these categories.^21^ Recently, overviews of PFAS use have emerged,^22^ while others have looked at strategies for grouping PFAS for the protection of human and environmental health^23^ or narrowed the OECD PFAS list down to those of commercial relevance, estimated to be ∼6% of the total list.^24^ Most, if not all, of these approaches are still largely manual.

While integrating the NORMAN-SLE content into PubChem,^6^ it became clear that the number of chemicals within PubChem (116 million chemicals, September 2023) that could satisfy the 2021 OECD PFAS definition dwarfed the several thousand entries in the common PFAS suspect lists. A simple substructure search for “CF_2_” revealed millions of potential matches in PubChem. Since new PFAS are emerging very rapidly, the need for a manageable, relevant, rapidly updateable open collection of PFAS for the community is increasingly obvious. This article describes efforts to develop an interactive, open, dynamic, and browsable collection of PFAS content in PubChem to serve this purpose.

## Materials and Methods

**Figure 1:**
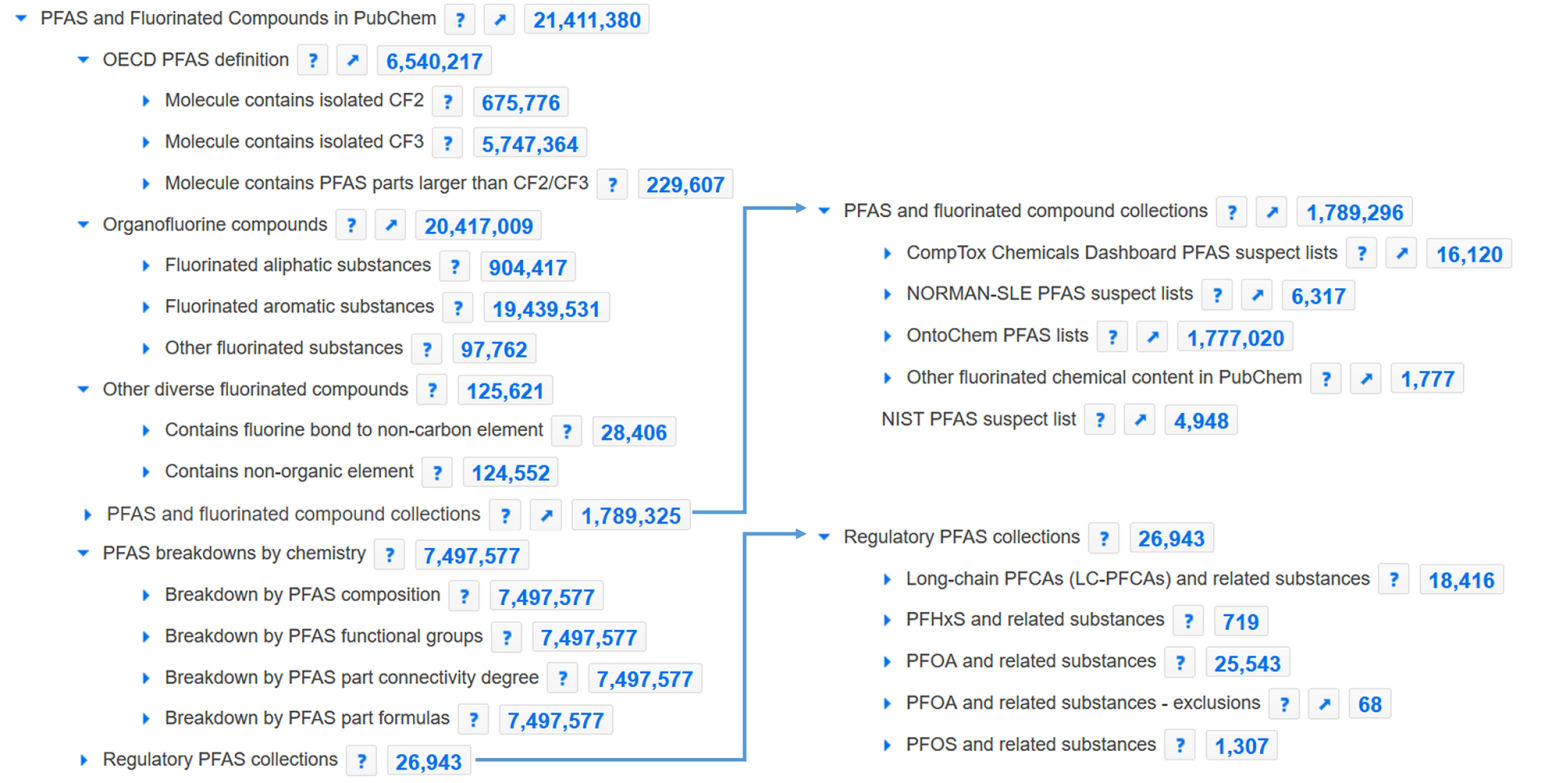
PFAS and Fluorinated Compounds in PubChem collection showing the six top nodes and the first layer of subnodes; collection available at https://pubchem.ncbi.nlm.nih.gov/classification/#hid=120. Image created September 16, 2023.

The “PFAS and Fluorinated Compounds in PubChem” collection (hereafter “PubChem PFAS Tree”) is openly available and is integrated into the Classification Browser of PubChem. It is designed to support the exploration and exchange of information about PFAS and fluorinated compounds within the community. This information is compiled and assembled using several different approaches, described in the following sections. The online collection (shown with the first two layers of nodes in Figure 1) is updated frequently and is available at https://pubchem.ncbi.nlm.nih.gov/classification/#hid=120.

### PFAS and Fluorinated Content in PubChem

Four sections of the PubChem PFAS Tree are collated by running custom-designed PERL scripts (available on GitLab^25^) over the entirety of PubChem on a weekly basis, since the chemical content of PubChem updates daily and annotation content weekly. The “*OECD PFAS definition”* section contains all discrete chemicals (excluding salts and mixtures) fulfilling the 2021 OECD PFAS definition^3^ quoted above (hereafter termed an “OECD PFAS”), while the “*PFAS breakdowns by chemistry”* section contains all discrete chemicals, including salts and mixtures, that are an “OECD PFAS”.^3^ Figure 8 of the OECD Monograph ENV/CBC/MONO(2021)25^3^ also included a breakdown of organofluorine content into several aliphatic and aromatic categories; this structure is reflected in the “*Organofluorine compounds”* section of the PubChem PFAS Tree (see Figure 1). Over 100000 fluorinated compounds in PubChem did not fit into the categories set out in the OECD Monograph, either because fluorine was connected to noncarbon atoms or because there was the presence of nonorganic elements (or both). These cases were separated into the “*Other diverse fluorinated compounds*” section, which was broken down into these two subsections (see Figure 1). A more detailed description of the contents of each section and how this is constructed are contained in the PubChem PFAS Tree documentation.^26^

The scripts that construct the PubChem PFAS Tree^25^ run over content that is publicly available. This data is found on the PubChem FTP site^27^ and via openly available active programming interfaces (APIs) such as PUG REST.^28,29^ The processing takes approximately 2 h to complete (processing each of the 337 structure data files, as of June 2023, in parallel) via the PubChem compute environment.

At this stage, the entire PubChem PFAS Tree is constructed across the compound space only; i.e., all entries within the tree are discrete chemicals that have a PubChem Compound Identifier (CID). Thus, polymers and UVCBs are not currently a part of the PubChem PFAS Tree (see Perspectives).

### Suspect Lists and Regulatory Collections in the PubChem PFAS Tree

The remaining two major sections of the PubChem PFAS Tree are compiled in a semiautomated manner using scripts in R and are integrated into construction of the entire PubChem PFAS Tree via mapping files. All code, mapping files, and associated supporting files are on the Environmental Cheminformatics (ECI) GitLab pages.^30^ These sections and code build likewise on publicly available PubChem functionality, some of which was custom designed to enable the work described here, including adding new classification browser functionality to PUG REST. The final integration of this content into the PubChem PFAS Tree is programmed and run in PERL, as part of the routine described in the previous section.^25^

“*PFAS and fluorinated compound collections*” contains five major sources of suspect lists (see top right inset of Figure 1), including NORMAN-SLE,^6^ CompTox,^11^ OntoChem,^18^ PubChem, and NIST.^15^ The CompTox chemical list content is retrieved programmatically from the PubChem EPA DSSTox Classification Browser^31^ (https://pubchem.ncbi.nlm.nih.gov/classification/#hid=105) and curated manually to retain only PFAS lists (41 lists as of 16 September 2023), which are included in the mapping file to retrieve the respective CIDs in each list via their classification hierarchy node identifier (HNID). The files containing the CIDs for the remaining four sources are hosted on the ECI GitLab pages; the URLs for each file are contained within the mapping file used for retrieval during the PubChem PFAS Tree construction. The NORMAN-SLE subsection contains all PFAS lists within the NORMAN-SLE (currently 12); one CID list was manually adjusted to remove non-PFAS entries, such as counterions. The OntoChem CID lists are broken down by the three PFAS definitions and two data sources to form six categories. The NIST PFAS Suspect List was downloaded and deposited to PubChem (resulting in 1232 new CIDs: i.e., new compound record entries in PubChem) and updated once all new CIDs were registered. Finally, the PubChem content was compiled by identifying several fluorinated compound sections in other classification browsers, including the MeSH, Cameo, and ChEBI browsers. These were also added by providing fixed files via the GitLab pages. These lists and mapping files are updated as necessary under full version control in GitLab;^30^ all updates appear with the next PubChem PFAS Tree update.

The final section, “*Regulatory PFAS collections*”, was added upon interactions with Andreas Buser from the Federal Office of the Environment (FOEN), Switzerland (see Acknowledgments), to support regulatory PFAS efforts. As shown in Figure 1, inset, bottom right, regulation surrounding four cases are covered: long-chain perfluorocarboxylic acids (LC-PFCAs), perfluorohexanesulfonic acid (PFHxS), perfluorooctanoic acid (PFOA), perfluorooctanesulfonic acid (PFOS), and the related substances for all cases. The fifth section deals with exclusions from the PFOA cases, which are separated to avoid “exclusions” being added to the PFOA category totals. Each section is constructed according to definitions from regulatory efforts such as the Stockholm Convention,^32^ European Union (EU) Registration, Evaluation, Authorisation and Restriction of Chemicals (REACH) and EU Environmental Chemicals Agency (ECHA).^33,34^ The sections include several lists published with these definitions as well as various PubChem queries to find matching content in PubChem according to the definitions. Exact details of the PubChem queries are in the respective tool tips (obtained by clicking the “?” next to each heading) and in the documentation.^26^ For the LC-PFCAs, the definitions came from reports UNEP/POPS/POPRC.17/7^35^ and UNEP/POPS/POPRC.18/6/Add.1^36^ as well as EU Regulation 2021/1297,^37^ with an indicative list from report UNEP/POPS/POPRC.18/INF/14.^38^ For PFHxS the definitions came from UNEP/POPS/COP.10/CRP.10^39^ and a draft ECHA report,^40^ while the initial indicative list came from UNEP/POPS/POPRC.15/INF/9.^41^ The definition for PFOA came from Annex A of the Stockholm Convention (2019 revision),^32^ while the initial, updated, and exclusions from the PFOA lists were taken from UNEP/POPS/POPRC.17/INF/14/Rev.1.^42^ Finally, the PFOS definition and PFOS listing were taken from Annex B of the Stockholm Convention.^32^ The motivation and methods behind these efforts are described further in the documentation,^26^ as well as in a presentation at POPRC.18^43^ and a webinar.^44,45^

## Results and Discussion

### Overview of PFAS and Fluorinated Compounds in PubChem

As shown in Figure 1, the number of fluorinated compounds (>21 million) and PFAS (7.4 million with salts and mixtures, 6.5 million without) in PubChem is much higher than the common PFAS screening lists of 4000–10000 entries. Of the 20 million organofluorine compounds classified according to the OECD^3^ (see Figure 1), ∼900000 are fluorinated aliphatic substances and 19.4 million are fluorinated aromatic substances; just under 100000 fall into the “other” category, which contain fluorine connected to noncarbon organic elements (a more detailed breakdown can be obtained by expanding the respective node in the PubChem PFAS Tree). Note that compounds can fall into more than one of these categories; the node totals always indicate the total number of CIDs under the entire node. For instance, there is no overlap between the fluorinated aliphatic and fluorinated aromatic substances, while 17067 of the “other fluorinated substances” are also “fluorinated aromatic substances” and 7634 are also “fluorinated aliphatic substances” (queries performed via PubChem “saved search” functionality on September 16, 2023). Approximately 120000 fluorinated compounds fall outside the OECD organofluorine classification,^3^ contained within the “*Other diverse fluorinated compounds*” node.

**Figure 2:**
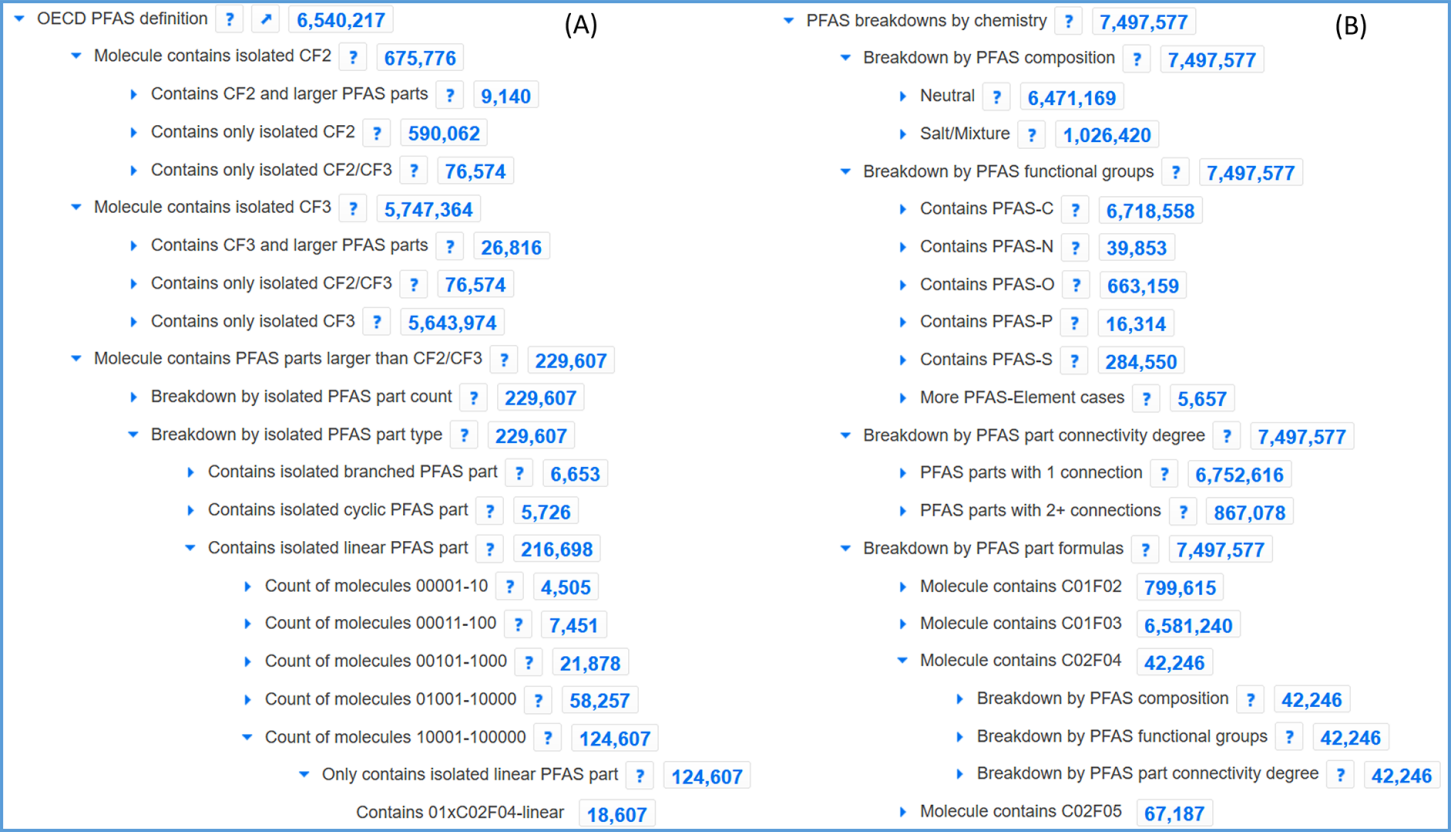
PFAS content according to the 2021 OECD definition (A) excluding salts and mixtures; (B) including salts and mixtures; (C) subnodes of the “Breakdown by PFAS functional groups” for PFAS-S; (D) Adding the keyword “PFAS-S(=O)2-N” to the search bar in the Classification Browser allows users to quickly find sulfonamide and related PFAS. Image created September 16, 2023.

A more detailed breakdown of the PFAS sections according to the updated OECD definition^3^ is shown in Figure 2. Figure 2A reveals that 6.5 million PFAS fit this new definition (excluding salts and mixtures), of which 5.7 million contain an isolated CF_3_ group, ∼670000 an isolated CF_2_ group, and ∼230000 a PFAS moiety larger than CF_2_/CF_3_—in other words, ∼230000 PFAS also satisfy the 2011 Buck et al.^2^ PFAS definitions of substances containing at least CF_2_–CF_2_. As shown in Figure 2A, this can be broken down further to determine, e.g., how many molecules with an isolated CF_3_ also contain larger PFAS parts (∼27000) and whether the parts are linear, branched, cyclic, and so on. As shown at the bottom of Figure 2A, the breakdown will eventually reveal the formulas of the PFAS part (here C_2_F_4_; note that the leading zeros are added to maintain a logical sorting order), should a given chain length be of interest. The total number of nodes in the tree is very high (9890 nodes in June 2023). The nodes below the major sections are created dynamically depending on the data to maintain performance and functionality. As a result, formulas and other nodes appear once certain conditions are met—more details are given in the documentation.^26^ Suspect lists and databases can be created for workflows by clicking on the nodes of interest (i.e., the blue numbers), which opens a search window to either browse or download the entries. The download file contains several fields of interest; details on how to perform searches and downloads are given further below and in the documentation.^26^

Figure 2B shows the breakdown of PFAS including salts and mixtures with ∼1 million additional entries due to salts and mixtures. The difference in numbers on the “OECD PFAS definition” total (6.54 million) versus the “Neutral” category (6.47 million, third row of Figure 2B) is due to differences in the processing as well as ambiguities in the wording of the PFAS definition. Currently, this difference is being maintained to enable an easier comparison of these “edge cases” (cyclic PFAS and PFAS-ether cases) and thus to stimulate discussion with experts within the PFAS community to help develop/refine PFAS definitions in a way that is both easy to understand and to implement consistently with automated cheminformatics approaches (discussed further below). Figure 2B also reveals additional ways of browsing the PFAS content in a complementary manner to Figure 2A, including by functional groups (with the PFAS part connected to C, N, O, P, S, or other elements), by connectivity (with only one connection, i.e., where the PFAS is a terminal part of the molecule, or with two or more connections to the PFAS part), and by formulas, so that it is possible to search by the length of the PFAS part if a particular chain length is of interest. Again, leading zeros are present in formulas to enable a logical sort order of the formulas since the classification browser nodes appear alphabetically. The section shown in Figure 2B can be broken down by each of the respective categories, such that it is possible to exclude salts and mixtures or only search for PFAS formulas connected to S, and so on. Figure 2C,D shows how to find, e.g., sulfonamide and related PFAS in the tree. The dynamic “*PFAS breakdowns by chemistry*” section (Figure 2B) contains 24600 nodes, over double the number of nodes in the “*OECD PFAS definition*” section (Figure 2A). Further details and examples are again given in the documentation^26^ and explained in the webinar.^44,45^

### Suspect Lists in the PubChem PFAS Tree

**Figure 3:**
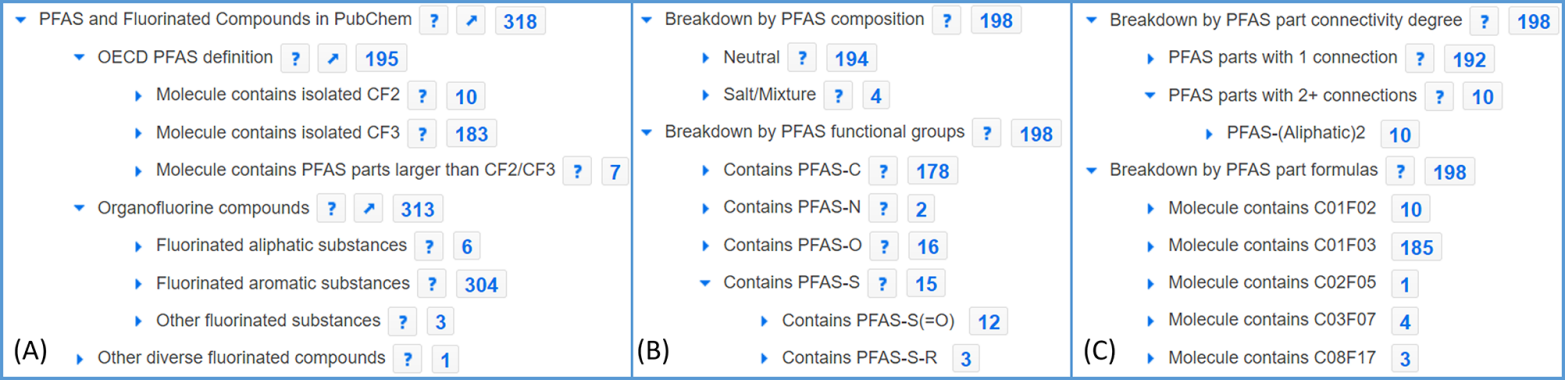
Exploring the contents of fluorine-containing pesticides, S94 FLUOROPEST,^48,49^ with the PubChem PFAS Tree: (A) OECD PFAS (195) and organofluorine content (313 of 318 entries—the missing entries are salts); (B) breakdown of PFAS (with salts), also showing heteroatom connections; (C) breakdown by connectivity degree and PFAS part formulas, revealing that most pesticides contain CF_2_ or CF_3_. Image created June 17, 2023, using PubChem Entrez functionality, explained further in the documentation.^26^

The suspect list section was entitled “*PFAS and fluorinated compound collections*” rather than “PFAS suspect lists” since the contents of various suspect lists were not always PFAS and extremely large lists such as the OntoChem Patent collection (>1 million entries) are too big for suspect screening. Most lists currently come from CompTox (41 entries as of September 16, 2023), including their PFASMASTER and PFASSTRUCTV5 lists. Each list can be downloaded individually, as for all nodes of the tree. While there is increasing interest in fluorine-containing pesticides and pharmaceuticals, not all entries in the published lists (e.g., lists S92^46,47^ and S94^48,49^ of the NORMAN-SLE, containing fluorinated pharmaceuticals^47^ and pesticides,^49^ respectively) are PFAS. By sending these nodes to PubChem Search and subsequently Entrez, it is possible to subset the entire PubChem PFAS Tree by a given suspect list (or combination thereof) and determine which entries are PFAS, organofluorine, etc., as shown in Figure 3. The steps required to perform this query are explained in greater detail elsewhere.^26,44,45^ The OntoChem lists, which are too big for efficient suspect screening, are already available elsewhere as database files.^50^ Note that the numbers in the suspect lists in the PubChem PFAS Tree may deviate from the original lists since only discrete chemicals are included, such that polymers and/or UVCBs will be missing (and the numbers consequently smaller) for lists containing polymer/UVCB entries in addition to discrete chemicals. Only one CompTox PFAS list (PFASMARKUSH) contained exclusively polymer/UVCB entries by design and is not displayed. While the OntoChem lists contained only discrete chemicals, these numbers also differ slightly from those of the published article^18^ due to edge cases encountered during PubChem deposition. As discussed in Barnabas et al.,^18^ different cheminformatics toolkits perceive the structures differently: PubChem uses internal code as well as the OEChem^51^ and CACTVS^52^ toolkits for standardization^53^ and deposition to create chemical records, while OntoChem uses OpenChemLib^54^ to produce their final lists.

The “*PFAS and fluorinated compound collections*” section is also designed to enable the addition of new PFAS or fluorinated content into PubChem as they are documented, to fill gaps in the database, and to ensure rapid discovery of new and relevant entries by the community. The necessity for a rapid discovery of new PFAS of concern is one motivation for the regular updates of the entire PubChem PFAS Tree. As mentioned above, the integration of these collections has resulted in the addition of >200000 new PFAS entries to PubChem, including >200000 from OntoChem, 1232 from the NIST PFAS Suspect List, and several entries from both the CompTox and NORMAN-SLE contributions, which have been deposited progressively over several years. Almost 25% of the NIST PFAS list was new content to PubChem, showing the importance of hand-curated expert knowledge from researchers to fill knowledge and database gaps. The NORMAN-SLE^6,7^ hosts several lists, developed using templates designed together with PubChem,^55,56^ which can be used to add new PFAS or other compounds as soon as a reference information is available, thus providing a channel for the scientific community to add new data to the public domain. Contact details are given in the documentation.^26^ Several examples of community contributions were provided in the webinar.^44,45^ The information should be available under an appropriate license (e.g., CC-BY^57^) to enable inclusion.

### Regulatory Collections

The final node in the PubChem PFAS Tree, “*Regulatory PFAS collections*”, allows users to investigate several aspects of PFAS regulation, including the impact of different wording in definitions under consideration on the number of compounds potentially covered by the regulation. The following paragraphs cover the different cases one by one. Further details on how to perform the search queries, overlaps, downloads and other functions mentioned below can be found in the tooltips, documentation,^26^ and webinar.^44,45^

The section “*PFOS and related substances*” is the simplest. It contains the original eight entries for “*PFOS plus salts, isomers and PFOSF*” listed in the Stockholm Convention Annex B^32^ and an extended listing of all content in PubChem matching the “*PFOS plus salts, isomers and PFOSF*” definition, currently 1307 entries in total (first node appearing in this section, which can be expanded to see the contributing subsections/categories). These 1307 entries comprise PFOS and branched isomers (18), PFOS, PFOSF, and salts (239), and a merged PFOS and PFOSF substructure query to find all matching mixtures (1290 CIDs). An additional section outlines compounds that transform to PFOS (under normal conditions, *i*.e., excluding advanced treatment transformations) that are in PubChem for information purposes, but these four entries are not included in the extended listing of “*PFOS and related substances*”.

**Figure 4:**
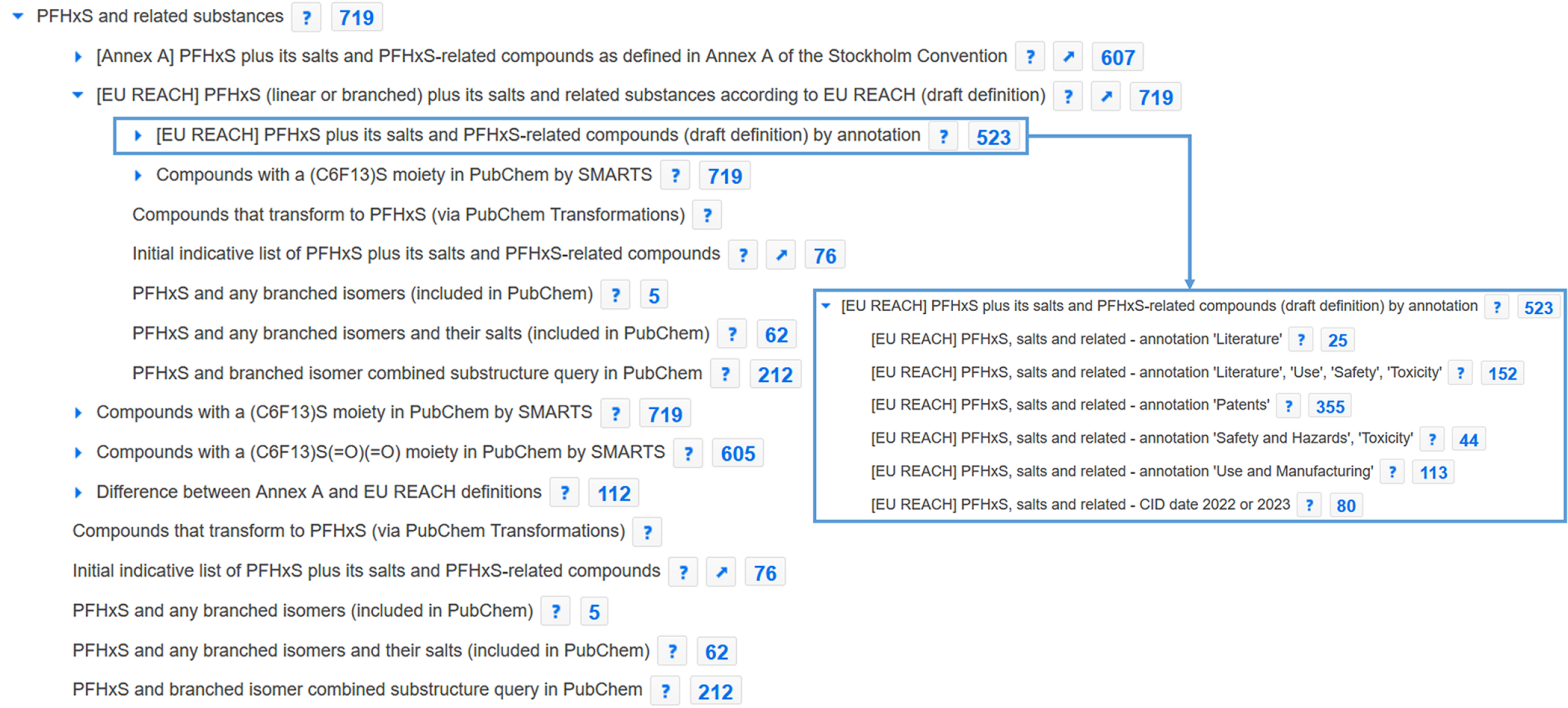
Regulatory collection: example of PFHxS with two different definitions (Annex A of the Stockholm Convention and a draft definition for EU REACH) from September 16, 2023. The main image shows how the node and EU REACH subnode are constructed; the inset shows the breakdown by annotation content to help find the most relevant (or recent) matches (also available for Annex A).

The “*PFHxS and related substances*” section contains a lot more detail than the PFOS section, as two different definitions are currently being explored for the Stockholm Convention and EU REACH. This is an interesting example, where a slight change in the wording of the definition results in a difference of over 100 CIDs (chemicals) in the resulting lists. The Stockholm Convention PFHxS definition^39^ defines related compounds as compounds with a C_6_F_13_S(=O)(=O) moiety (605 CIDs in total), whereas the EU REACH definition^40^ defines this as C_6_F_13_S (719 CIDs total). Both definitions appear at the top of the PFHxS section, with content breakdowns (indicated by blue arrows in Figure 4) to show how these have been compiled.

For each PFHxS definition, a breakdown by major categories of annotation content has also been provided (see inset of Figure 4 for the example of EU REACH), including whether literature, use and manufacturing, safety and hazards, toxicity, or patent information is available in PubChem, or whether the chemical was added only recently (CID date 2022 or 2023). In total, 607 CIDs are covered under the Stockholm Convention PFHxS definition,^39^ 312 with patent, 108 with use, 43 with safety or toxicity, 15 with literature information, and 76 recent entries (from 2022 or 2023). The EU REACH definition contains 719 CIDs total, 355 with patent, 113 with use, 44 with safety/toxicity, 25 with literature information, and 80 recent CIDs (see Figure 4 inset). The section exploring the difference between the definitions contains 112 CIDs in total, of which relatively few have either use, literature, or safety/toxicity information (only 14 CIDs total).

Although PFOA, like PFOS, has been regulated already for several years, the PFOA section was much trickier to construct than the PFOS section and remains incomplete due to the wording of the definition in Annex A of the Stockholm Convention.^32^ The entire node currently contains 25543 CIDs, but only 789 of these have been included in the “*PFOA plus its salts and PFOA-related compounds as defined in Annex A of the Stockholm Convention*” section, since the exclusions to the definition are almost impossible to define or automate cheminformatically with the existing PubChem functionality. Thus, at this stage, entries that (to the best of our knowledge) meet the definition have been included, and several other sections are included under this node for users to explore other content further. The entries that are included are the selected and updated lists from the Stockholm Convention^32^ (80 and 299 CIDs, respectively) plus three PubChem queries covering PFOA and branched isomers (47 CIDs), PFOA, branched isomers and salts (162 CIDs), and the PFOA plus branched isomer substructure query to capture mixtures (546 CIDs). An additional section breaks down the 789 matching PFOA content by annotation categories, such as those found in the literature (81), use information available (228), safety or toxicity information (41), patent information (402), or recent addition (in 2022 or 2023, 60 entries). This helps to find potentially relevant entries among the hundreds of potentially regulated matches. The PFOA exclusions have been included in the node below with placeholder nodes for content that cannot currently be created with reasonable effort. The halide exclusions have been implemented (currently 26 entries), and the updated indicative list of exclusions has been provided (35 CIDs). Polymers are inherently excluded from the tree, as it currently covers compound space only, with additional functionality to enable polymer/UVCB inclusion still under active development at PubChem (and thus a potential future extension). The automatic detection of the remaining two exclusion categories, perfluoroalkyl carboxylic and phosphonic acids (including their salts, esters, halides, and anhydrides) with ≥8 perfluorinated carbons, plus the perfluoroalkanesulfonic acids (including their salts, esters, halides, and anhydrides) with ≥9 perfluorinated carbons, has proven tricky. Although it may theoretically be possible to implement these exclusions programmatically, the current wording would require the creation of thousands of lines of custom code or several hundred very inefficient queries, which, given the potentially thousands of possible matching entries, would be likewise difficult to check for accuracy and curate accordingly (several attempts at implementing this have been made already and sidelined as currently unviable). This remains an area of development for the PubChem PFAS Tree and a conversation topic with regulators, highlighting the challenges in implementing the current definition into an automated cheminformatics workflow, which will be necessary to update these regulatory lists in a manner that is scalable to the current numbers of PFAS (millions).

Like PFOA, the LC-PFCAs section remains difficult to complete due to the sheer number of chemicals involved. This is primarily due to the wording choice in the definition for the “related chemicals”. As for PFHxS, two definitions are being explored for LC-PFCAs, the Stockholm Convention nomination of C_9_–C_21_ LC-PFCAs^35^ and the EU REACH definition of C_9_–C_14_ LC-PFCAs.^37^ The CIDs contained within these sections currently fulfill the LC-PFCAs, branched isomers, salts, and mixture requirements of the regulation but have not been extended to the related substances which, even in the current incomplete state, cover an additional 18416 entries (the “related substances” subsection remains as work in progress as the functionality required to perform these queries efficiently and automatically is still being developed). The C_9_–C_14_ LC-PFCAs section is constructed using the “*PFAS breakdowns by chemistry*” section of the PubChem PFAS Tree and contains 229 CIDs. The C_9_–C_21_ LC-PFCAs section contains 745 CIDs, which includes the draft indicative listing (83 CIDs), compounds that transform to LC-PFCAs (3 CIDs), plus queries for C_9_–C_21_ LC-PFCAs, their branched isomers, salts, and mixtures. In total, 584 of these have some form of annotation content, including 129 with use, 34 with safety or toxicity, 47 with literature, 490 with patent information, and finally 38 CIDs created recently (from 2022 or 2023). Again, these categories help determine which of the C_9_–C_21_ LC-PFCAs may be relevant for different use cases.

All of the numbers presented in this section that have been created via PubChem queries will potentially shift with updates (most likely increasing) as the content in PubChem changes and grows.

### Interacting with the PubChem PFAS Tree

The number of PFAS contained within the PubChem PFAS Tree, let alone the number of fluorinated compounds, is overwhelming. As mentioned in previous sections, there is a large amount of data present to add context to these numbers, as well as a variety of search functions and workflows to browse, explore, and subset the contents further to help find the most relevant PFAS or fluorinated compounds for given use cases. This section gives a brief overview of some possibilities, with further information available in the PubChem documentation,^58^ PubChem PFAS Tree documentation,^26^ and the webinars.^43−45^

**Figure 5:**
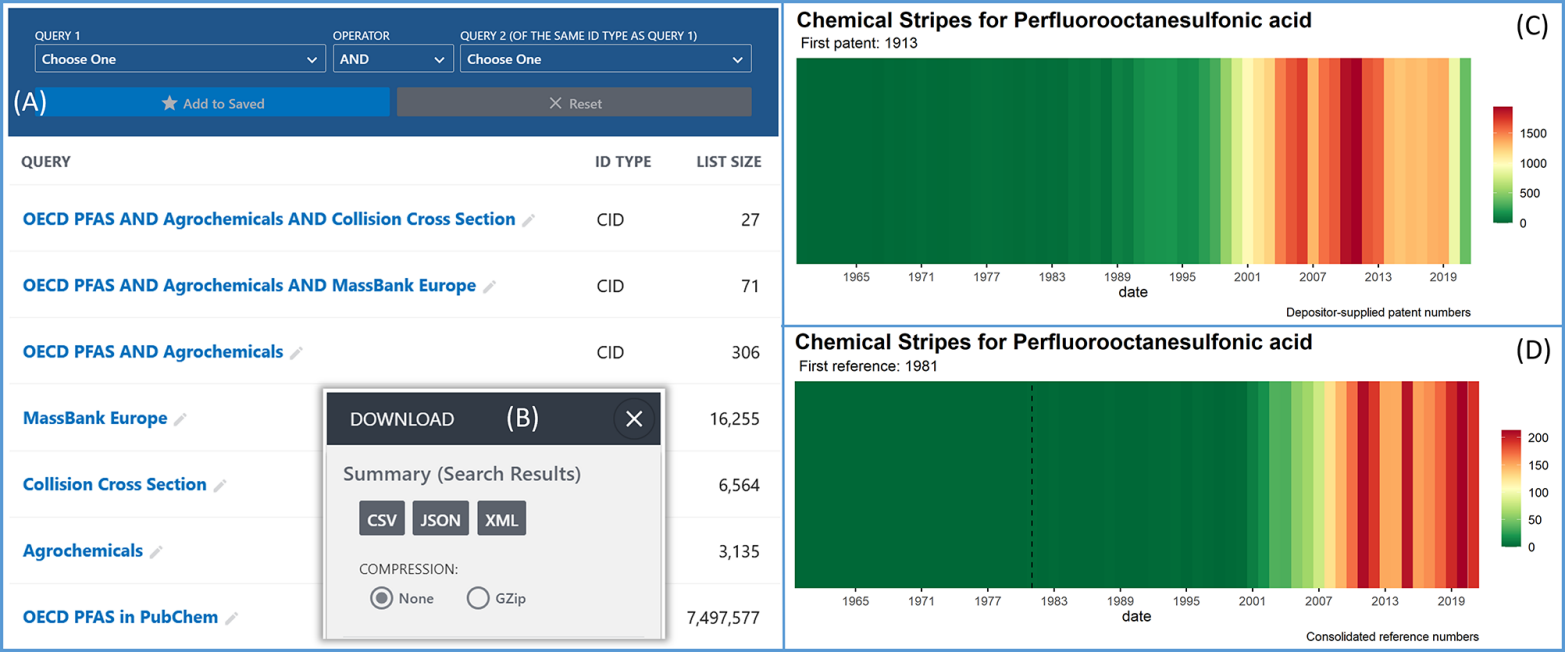
Interacting with annotation information associated with the PFAS content in PubChem. (A) The “saved searches” panel, which allows exploration of the overlap (AND, OR, NOT) between various categories and searches via the blue panel at the top (query names can be edited). (B) Any search, or combination of searches, can be downloaded in a variety of formats (download fields described in the text). (C) Chemical stripes^60−62^ on the patent data for PFOS, extracted from the PubChem patent tables. (D) Chemical stripes for PFOS based on consolidated reference values, extracted from PubChem Consolidated References tables.

Every node in the PubChem PFAS Tree (i.e., the blue numbers besides each category name in Figures 1–4) or any classification browser in PubChem can be sent to PubChem Search by clicking on the numbers. A separate search window will open, which allows browsing and sorting of the results, the ability to interact with individual compound records, as well as the ability to save and combine searches (see Figure 5A) or send the content to Entrez for advanced search building and/or to browse in the classification browser (see Figure 3 for example outputs). Each search query can then be downloaded in a variety of formats (Figure 5B). It is also possible to upload custom lists to search via the PubChem landing page^20^ (either pasting into the search bar, or via the “Upload ID list” option) or the PubChem Identifier Exchange.^59^ The “Keyword” field in the Classification Browser can be used to perform text searches on nodes of the tree (see the example in Figure 2D).

The download file contains several useful fields, a selection of which will be described here (for more information and a figure, see the documentation^26,58^). Names, synonyms (including CAS numbers where provided), identifiers (PubChem CID, the International Chemical Identifier (InChI),^63^ and the hashed form InChIKey^63^), and structures in the Simplified Molecular Input Line Entry System (SMILES)^64,65^ format are included. Several property fields are also given, including molecular formula, exact mass, molecular weight, and predicted octanol–water partitioning coefficient (XlogP^66^). Several additional fields help add context to the chemicals, including (at the time of writing; column header in brackets) the consolidated literature count (pclidcnt), patent count (gpidcnt), annotation categories (annothits), the count of annotation (annothitcnt), the date the CID was added (cidcdate), the names of the sources who deposited this structure (sidsrcname), and the deposition categories of the sources (depcatg). The annotation categories will be discussed more in the next paragraph; note that the columns, headers, and content are potentially subject to change. The patent and literature counts have been used for many years to help prioritize chemicals in nontarget identification efforts,^67^ but as demonstrated in Figure 5C,D, the distribution of the counts shown by the Chemical Stripes^60−62^ per chemical can also reveal interesting patterns, with the patent data often increasing earlier than the literature. This means that patent data could potentially be useful to find chemicals that are being used increasingly in industry (above the trend of other chemicals) before they are discovered through problematic emissions.^68^ It is possible to find recently added CIDs using the CID date (cidcdate). Since PubChem originated in 2004, this CID date will not always be an accurate reflection of the origin date of older chemicals. For older chemicals, the literature and patent dates can help build a more accurate history, as shown in Figure 5C,D for PFOS, which was first added to PubChem in 2005 but was first mentioned in patents in 1913 and in the literature (within the collection available to PubChem) in 1981. The name of the depositors and the deposition category can help distinguish whether these chemicals come exclusively from patent literature or combinatorial libraries used for drug discovery or whether these have been deposited by researchers, the US EPA, and so on. While these lists can be extremely long for well-known PFAS, these also tend to have substantial quantities of annotation, literature, and patent counts; the source information can help distinguish interesting entries among the long tail of matching chemicals with very little other data that potentially include chemicals of high concern that have only just been discovered and documented.

The annotation content of PubChem is very rich, coming from a wide variety of sources (currently over 930 data sources contribute to PubChem). The download file contains information on several major categories. The most relevant ones for environmental applications include, for example: drug and medication information; food additives and ingredients; literature; patents; pharmacology and biochemistry; safety and hazards; toxicity; use and manufacturing. The presence of these categories in the download file makes it easy to filter the results by the categories of interest. Further annotation content can be browsed using the PubChem Table of Contents (TOC) Classification Browser (the “landing page” of the classification browser at https://pubchem.ncbi.nlm.nih.gov/classification/#hid=72), which provides an overview of all annotation content in PubChem, currently 603 categories (September 16, 2023). The overlap of PFAS and annotation content can be explored using the PubChem saved search and Entrez functionality. Figure 5A demonstrates how the “saved search” feature can be used to calculate how many OECD PFAS (7497577 CIDs, bottom row) are also agrochemicals (from the TOC heading, 3135 CIDs, of which 306 are also OECD PFAS, third row) with mass spectral data in MassBank Europe (second row: 71 CIDs that are OECD PFAS agrochemicals in MassBank Europe) or measured collision cross section (CCS) data (top row: 27 CIDs that are OECD PFAS and agrochemicals with experimental CCS values in PubChem). Each of these overlap queries can also be browsed/downloaded. Further information on how to perform these queries is available in the PubChem documentation,^58^ PubChem PFAS Tree documentation^26^ and in the webinars.^43−45^

### Perspectives

Creating a dynamic, user-friendly, browsable, and intuitive resource to explore >21 million fluorinated compounds in PubChem has been an incredibly challenging exercise in informatics and design, with several draft approaches attempted and revised before settling on the current version presented here. The functionality remains under development; automation of the regulatory and suspect list sections will be improved as the required functionality is developed. The handling of PFAS ethers (CF_2_–O connections) and cyclic PFAS structures has been particularly challenging, along with the implementation of automated queries for the PFOA exemptions and the related compounds for the LC-PFCAs (as described above). While salts and mixtures have been added to the OECD PFAS section (resulting in an extra million CIDs included in the PubChem PFAS Tree), these are still missing in the “*Organofluorine compounds*” and “*Other diverse fluorinated compounds*” sections. With rising awareness of fluorinated counterions increasing in concentrations in wastewater and potentially becoming problematic for treatment and thus drinking water production,^69^ adding this is a shorter term future development, which may add a few million more CIDs to the PubChem PFAS Tree. Polymers and UVCBs will be added to the PubChem PFAS Tree once PubChem functionality is available to do so and will likewise increase numbers further.

Community feedback has been and will continue to be valuable to help improve the design and features of future versions, potentially including the addition of new sections or substantial revision of existing sections where this is justified. Suggested future additions include a “%F content” definition such as that used in the CompTox PFASSTRUCTV5 list. The addition of the annotation content breakdowns to the regulatory collection was based on many questions from users about how to find the most relevant PFAS entries. As this annotation content is also available in the download files, it is possible to retrieve this information for any subset of the PubChem PFAS Tree using the various features described above. Although it is currently not possible for users to filter by annotation content, this will be considered in future PubChem developments. However, since the annotation data in PubChem are compiled from publicly available data and user contributions, it is not completely exhaustive. In other words, the presence of “Use and Manufacturing” information for a PFAS implies that this information is available in PubChem for that chemical with a suitable reference, but this does not imply that the entire “Use and Manufacturing” section covers all known uses.

The PubChem PFAS Tree has been available since March 2022, was the subject of several presentations and webinars,^43−45^ and has already been used in published research.^70^ Contributions of new PFAS or fluorinated chemicals and/or related annotation content, as well as feedback and suggestions about how the PubChem PFAS Tree can help the PFAS community answer their pressing questions, are very welcome.

## Data Availability

The raw data (SDF) are publicly available from the PubChem FTP site, and code (R, Perl) is available on GitLab.^25,30^ This material was submitted as a preprint on ChemRxiv: Schymanski, E. L.; Zhang, J.; Thiessen, P. A.; Chirsir, P.; Kondic, T.; Bolton, E. E. Per- and polyfluoroalkyl substances (PFAS) in PubChem: 7 million and growing. 2023. ChemRxiv. 10.26434/chemrxiv-2023-j823z (accessed September 15, 2023).

## Supporting Information Available

Detailed documentation, with current version available on GitLab (RMarkdown) and as a PDF^26^ (PDF)
