# Supplementary material for: Per- and Polyfluoroalkyl Substances (PFAS) in PubChem: 7 Million and Growing
Source: Environ Sci Technol. 2023 Oct 23;57(44):16918–28. doi: 10.1021/acs.est.3c04855 (PMC10634333; doi:10.1021/acs.est.3c04855)
Supplement: Supplementary file 1 — es3c04855_si_001.pdf [file es3c04855_si_001.pdf]

PFAS and Fluorinated Compounds in PubChem Tree

Emma L. Schymanski[1]*, Parviel Chirsir[1], Todor Kondic[1],
Paul A. Thiessen[2], Jian Zhang[2] and Evan E. Bolton[2]*

11 September 2023

[1] Luxembourg Centre for Systems Biomedicine (LCSB), University of Luxembourg, 6 avenue du Swing, 4367, Belvaux, Luxembourg. *ELS: emma.schymanski@uni.lu. ORCIDs: ELS: 0000-0001-6868-8145, PC: 0000-0002-9932-8609, TK: 0000-0001-6662-4375.

[2] National Center for Biotechnology Information (NCBI), National Library of Medicine (NLM), National Institutes of Health (NIH), Bethesda, MD, 20894, USA. *EEB: evan.bolton@nih.gov. ORCIDs: PAT: 0000-0002-1992-2086, JZ: 0000-0002-6192-4632, EEB: 0000-0002-5959-6190.

Preamble

This document describes the "PFAS and Fluorinated Compounds in PubChem Tree" (hereafter "PubChem PFAS Tree") in PubChem [1], developed jointly between PubChem (NCBI/NLM/NIH) and the Environmental Cheminformatics group (ECI) at the LCSB, University of Luxembourg, in consultation with several community representatives (see Contributions and Acknowledgements). The PubChem PFAS Tree (see Figure 1 and Contents listing) includes all compounds in PubChem satisfying various definitions, as explained later in this document. Note that each compound in PubChem has a PubChem Compound Identifier (CID), and the blue numbers next to each node header reflects the number of compounds (*i.e.* CIDs) in that node.

More details on the general PubChem Classification Brower features are given in the Section Exploring the Tree, via the PubChem documentation and help pages, or by reaching out to pubchem-help@ncbi.nlm.nih.gov for more information. Further information includes two videos on the ZeroPM YouTube channel, a ~23 min interactive walkthrough (Jun. 2022) and a ~1 hour webinar (Mar. 2023) [2], plus a preprint on ChemRxiv [3].

Contents

Table 1: *Contents list for the PubChem PFAS Tree documentation.*

Section	Navigation	PDF Page
PubChem PFAS Tree Nodes	Go to heading	2
- *OECD PFAS Definition*	Go to heading	2
- *Organofluorine Compounds*	Go to heading	5
- *Other Diverse Fluorinated Compounds*	Go to heading	6
- *PFAS and Fluorinated Compound Collections*	Go to heading	7
- *Regulatory PFAS Collections*	Go to heading	7
- *PFAS Breakdowns by Chemistry*	Go to heading	9
Exploring the PubChem PFAS Tree	Go to heading	10
- *Download via PubChem Search*	Go to heading	10
- *PubChem Saved Searches*	Go to heading	11
- *Interactions via Entrez*	Go to heading	12
Extra Details	Go to heading	15
Statements and References	Go to heading	16

PubChem PFAS Tree Nodes

The tree is currently split into six main nodes that are constructed and compiled separately (see Figure 1). Nodes that are under development are released once they are ready. Further details about each of the nodes are given below. PubChem Classification Browser features are described further in the Section Exploring the Tree.

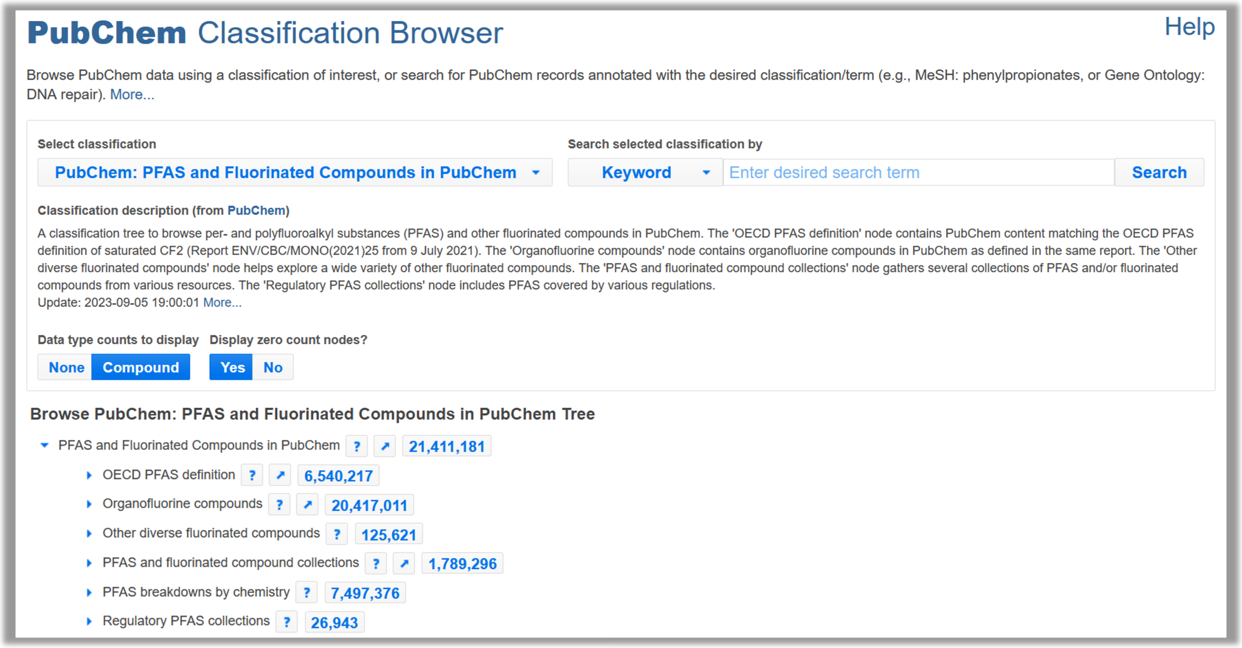

Figure 1: *The "PFAS and Fluorinated Compounds in PubChem Tree" Landing Page (11 Sept. 2023).*

OECD PFAS Definition

This node is constructed out of per- and polyfluoroalkyl substances (PFAS) satisfying the OECD 2021 definition (contains at least one saturated CF_2 or CF_3 part) in the 2021 OECD Report ENV/CBC/MONO(2021)25 [4]. Note that here, "**PFAS part**" is used to describe a connected portion of the molecule that satisfies the OECD PFAS definition. A given molecule may have more than one PFAS part present, some examples are given in Figure 2, along with the count of parts.

Browsing the >6 million entries in this node (see Figure 3) is challenging. Since most of these PFAS contain isolated CF_2 (>670 K entries) or CF_3 groups (>5.7 M entries), these were separated into individual sections (see "Isolated CF_2 and CF_3 Nodes"). Approximately 229 K compounds contain PFAS parts larger than CF_2/CF_3 (see "PFAS Parts Larger than CF_2/CF_3").

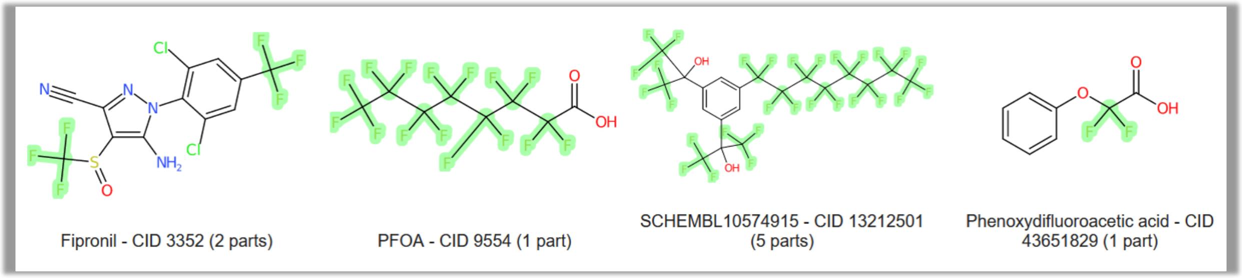

Figure 2: *Examples of molecules with varying PFAS parts highlighted, drawn using CDK Depict [5].*

The *OECD PFAS Definition* node, with the top two level subnodes, is shown in Figure 3.

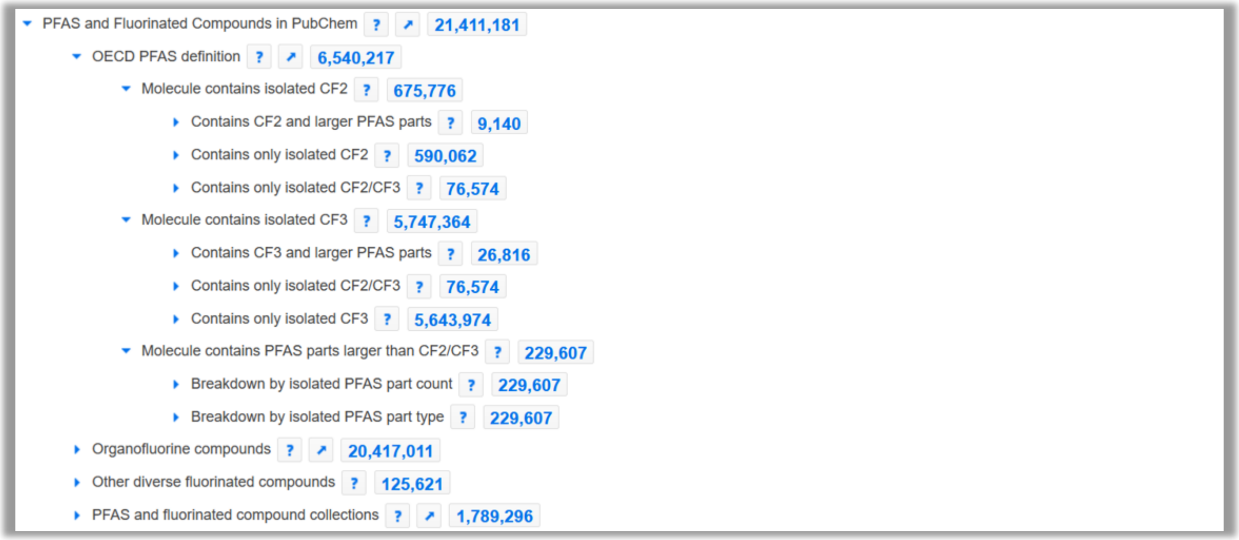

Figure 3: *The OECD PFAS Definition part of the PFAS tree, with top two subnodes (11 Sept. 2023).*

OECD PFAS - Isolated CF_2 and CF_3 Nodes

The *Isolated CF_2 and CF_3* subnodes of the *OECD PFAS Definition* node allows the browsing of all PFAS molecules in PubChem containing at least one isolated CF_2 (top subnode) or one isolated CF_3 (next subnode). These are broken down similarly, as shown in Figure 4 for CF_2.

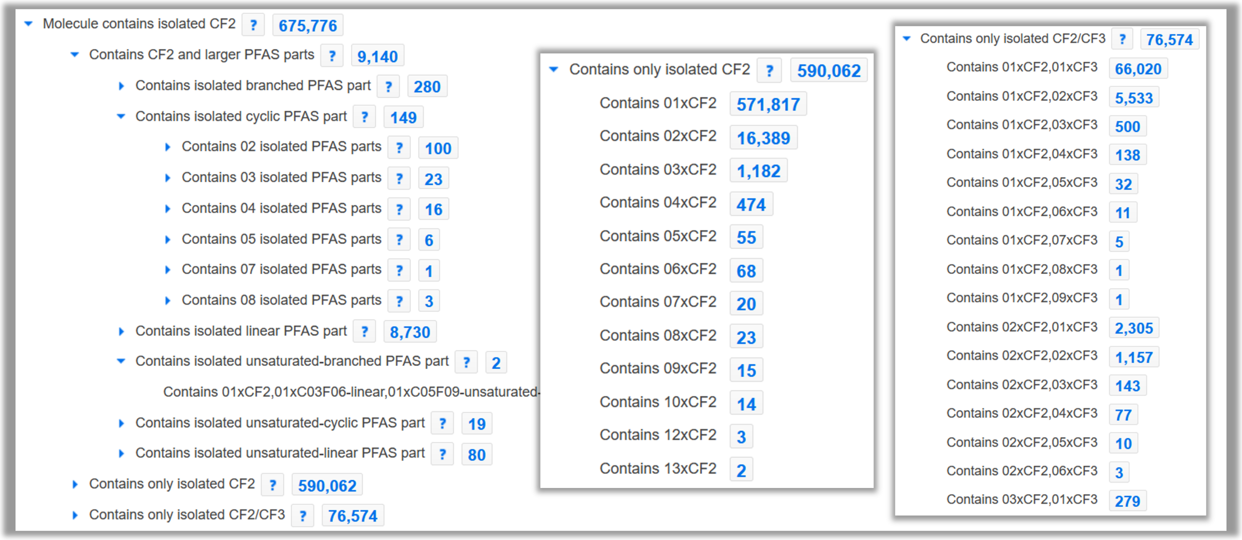

Figure 4: *The isolated CF_2 section of the OECD PFAS Definition node, with breakdown of the major parts (numbers as of 11 Sept. 2023).*

The larger PFAS parts (left) are broken down by part type (linear, branched, *etc.*). Within these subcategories, dynamic construction is used. If many (>20) variants are present, a breakdown by number of PFAS parts is added (*e.g.*, Figure 4, middle left, "*Contains isolated cyclic PFAS part*"), if not, a list of the possibilities is given directly (*e.g.*, Figure 4, lower left, "*Contains isolated unsaturated-branched part*").

The "*Contains only isolated CF_2*" (or, for the CF_3 node, "*Contains only isolated CF_3*") is broken down by the number of isolated groups (CF_2 or, for the CF_3 node, by CF_3 groups) - see Figure 4, middle panel. In both cases, the vast majority of molecules have only one isolated group. The "*Contains only isolated CF_2/CF_3*" node is also broken down by the number of groups, sorted by increasing number of CF_2 groups (for both nodes). See Figure 4, right panel.

OECD PFAS - PFAS Parts Larger than CF_2/CF_3

The "*Molecule contains PFAS parts larger than CF_2/CF_3*" part of the OECD PFAS node includes >220 K molecules, which can be browsed in two major breakdowns, by *isolated PFAS part count* (see Figure 5) and by *isolated PFAS part type* (see Figure 6). This section of the tree is constructed dynamically - in other words, the subnodes present depend on the contents within - to prevent excessive scrolling.

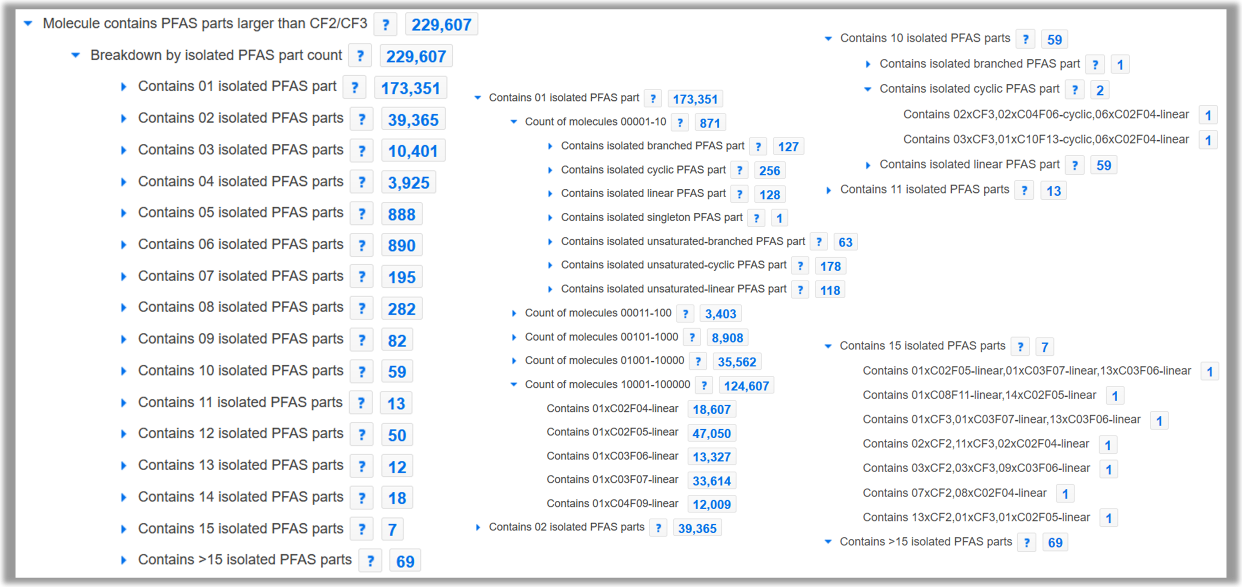

Figure 5: *The "Molecule contains PFAS parts larger than CF_2/CF_3" part of the OECD PFAS Definition node, with dynamic breakdown of subnodes by isolated PFAS part count (numbers from 11 Sept. 2023).*

The *Breakdown by isolated PFAS part count* is first subset by the number of parts (Figure 5, left panel). Should there be fewer than ~20 categories, the immediate breakdown is by the formula of the parts (see *e.g.,* Figure 5, bottom right, "*Contains 15 isolated PFAS parts*"). Should there be more than 20 entries, an extra layer is added, to sort by the type of PFAS part (see Figure 5, top right, "*Contains 10 isolated PFAS parts*"). For categories with very large numbers of entries, an additional initial breakdown by the count of molecules is added (Figure 5, middle panel). This is again broken down dynamically. If only a few subcategories exist, these are presented immediately thereafter (see Figure 5, bottom middle - several linear categories with many molecules). If, however, more breakdown is needed, an additional set of part type nodes is added (*e.g.,* Figure 5, middle panel, "*Count of molecules 00001-10*") before the formula breakdown. Note that throughout the tree, leading zeros are present to ensure logical sorting.

The *Breakdown by isolated PFAS part type* is first broken down by the part type (linear, cyclic, *etc.*) as shown in Figure 6, left panel. These are again split dynamically. With fewer than 20 entries, the list split according to PFAS part formulas appears. If a greater breakdown is needed, an extra layer of "*Also contains ...*" or "*Only contains ...*" is added for extra navigation (*e.g.,* Figure 6, mid left, "*Contains isolated branched PFAS part*"). For entries containing many CIDs, a breakdown by count of molecules is added (*e.g.,* Figure 6, mid left, "*Contains isolated linear PFAS part*"). Generally, the linear entries contain more entries than the other PFAS part types - and thus tend to have greater breakdown. Some of these are

broken down further (*e.g.,* Figure 6, right), such that a breakdown by the count of PFAS parts is added before the breakdown by "*Also contains. . .*".

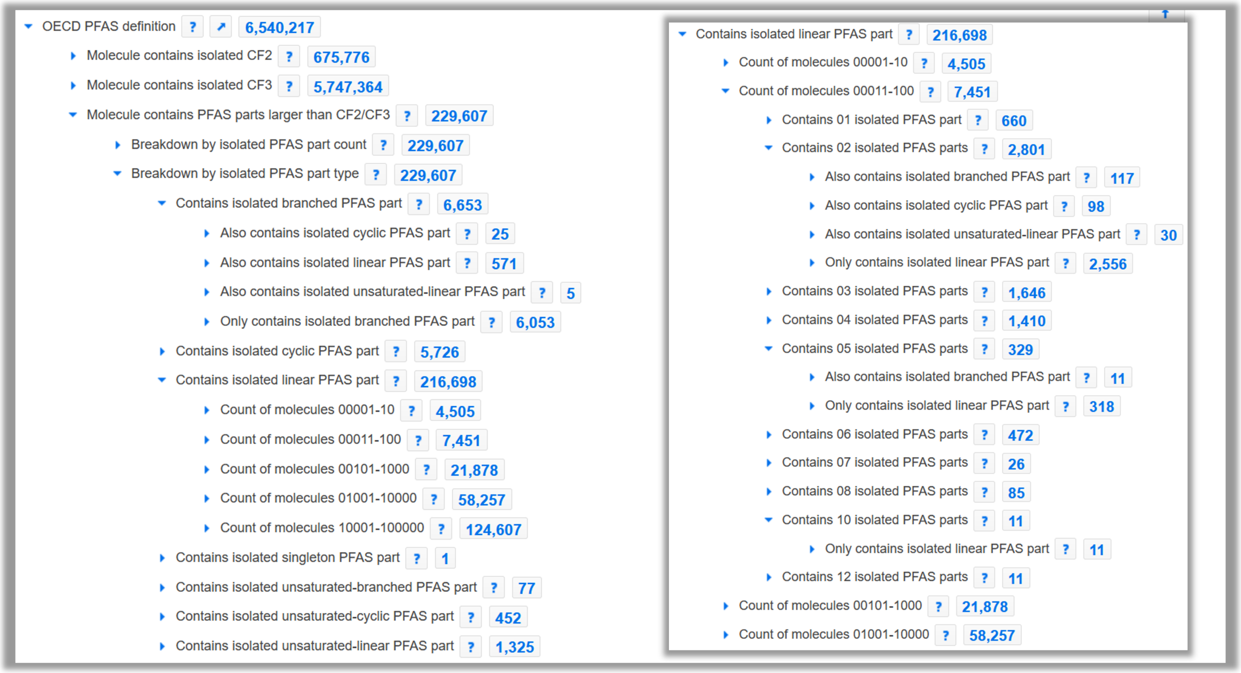

Figure 6: *The "Molecule contains PFAS parts larger than CF_2/CF_3" part of the OECD PFAS Definition node, with dynamic breakdown of subnodes by isolated PFAS part type (numbers from 11 Sept. 2023).*

The dynamic navigation approach reduces the scrolling by users and also helps reduce the data loading time when many entries are present within a node. It is possible to use some advanced search and querying capabilities to improve the interaction with the tree, see examples in Exploring the Tree below.

The *PFAS Parts Larger than CF_2/CF_3* is available as a MetFrag [6] file for further use [7]. The CSV can be downloaded from Zenodo (DOI: 10.5281/zenodo.6385954) for use in MetFragCL and is available from the MetFragWeb dropdown menu. This file contains several useful fields from the Download file as well as Patent and Literature (PMID) counts. See the description on the Zenodo record [7] for more details.

Organofluorine Compounds

This node contains *Organofluorine compounds* as defined in Figure 8 in the 2021 OECD PFAS Report ENV/CBC/MONO(2021)25 [4]. Figure 7 (below) shows an extract from Figure 8 of the OECD report on the left panel, and the corresponding node breakdown in the *Organofluorine compounds* section of the PubChem PFAS Tree to the right. Note that one additional category was added ("*Other fluorinated substances*") to capture content that did not fit into any other category defined in Figure 8 from the OECD Report.

The *Organofluorine compounds* node is broken down very differently to the *OECD PFAS Definition* node, since not all the contents are PFAS (and thus do not contain PFAS parts). Each subnode is broken down first by the number of fluorine atoms (1 through to 15, then >15) and then by an exact mass range. If there are no CIDs for the given category, it is not present. For instance, the "*Fluorinated aliphatic substances that have a fully fluorinated methyl or methylene carbon atom*" category starts at "*Contains 02 Fluorine atoms*" as no entries in this category could contain only one F. The exact mass subcategories are split into the ranges 1-250, 250-500, 500-750, 750-1000 and >1000 - and are only present if there are CIDs within this range.

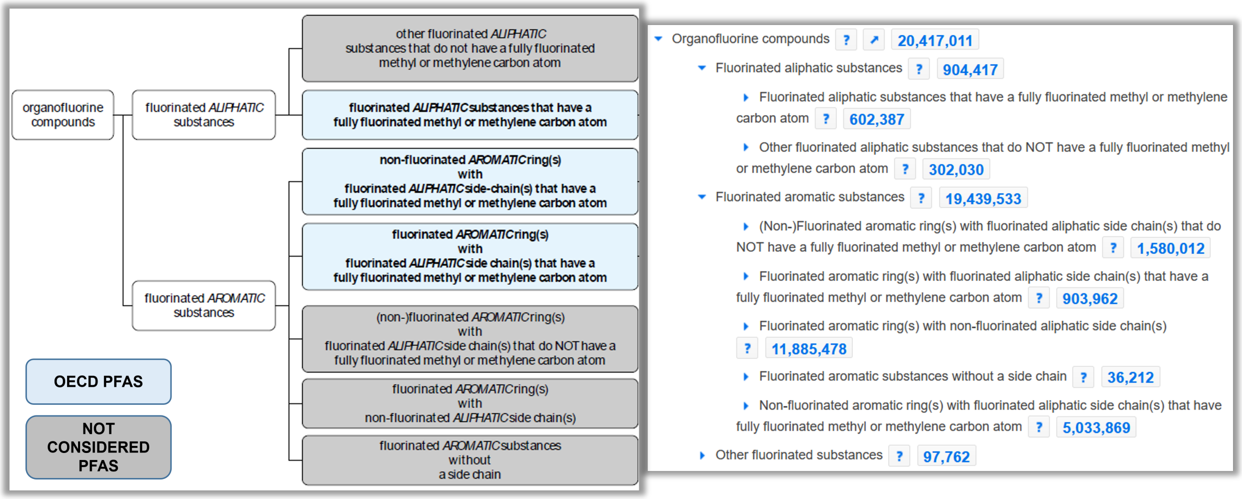

Figure 7: *The categorization of PFAS (blue shading) and non-PFAS (grey) from the OECD 2021 report [4] (left panel) and the "Organofluorine compounds" node (right panel). Numbers from 11 Sept. 2023.*

Other Diverse Fluorinated Compounds

The "*Other Diverse Fluorinated Compounds*" section of the PubChem PFAS Tree is designed to help users explore various cases of fluorine chemistry that are not necessarily covered in the OECD PFAS or Organofluorine compound sections above. The navigation in this section helps explore fluorinated compound chemistry by various fluorine-heteroatom bonds and the occurrence of different elements (see Figure 8).

Many of the compounds present in this section are also present in the other sections of the PubChem PFAS Tree. The overlap can be investigated in Entrez (see section Interactions via Entrez below).

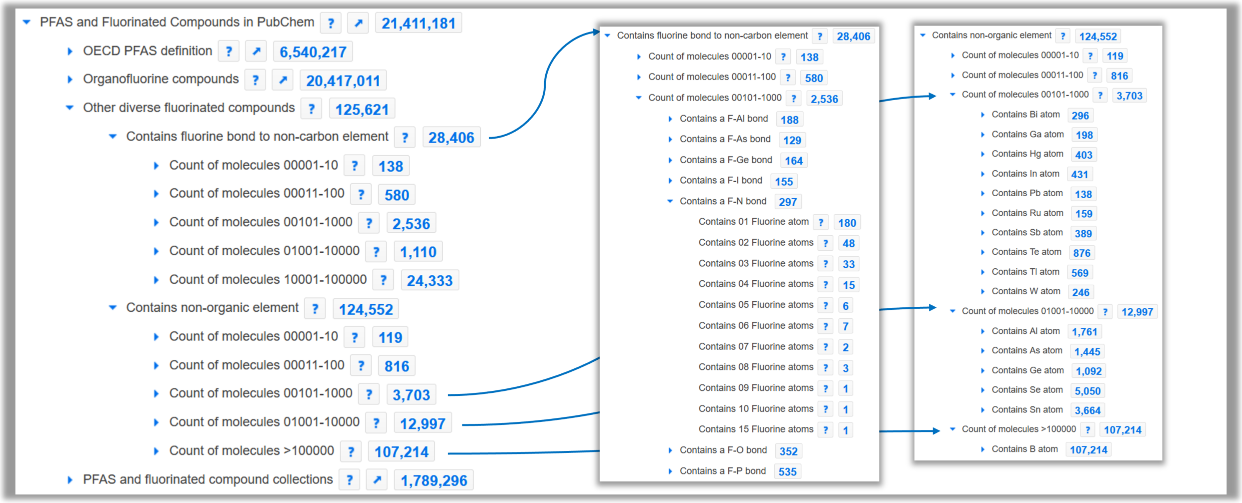

Figure 8: *The "Other diverse fluorinated compounds" part of the PubChem PFAS Tree, showing the breakdown by fluorine bonded to non-carbon elements and by non-organic element. Numbers from 11 Sept. 2023.*

The *Contains fluorine bond to non-carbon element* section (Figure 8, middle panel) is broken down first by the count of molecules present in the given category, then by the non-carbon element present in the F-element bond (sorted alphabetically). For the sections with counts above 100, there is an extra breakdown by the numbers of fluorine present overall.

The *Contains non-organic element* section (Figure 8, right panel) is likewise broken down first by the count of molecules present in the given category, then by the non-organic element present (sorted alphabetically). In this section, non-organic refers to any element that is not C, H, N, O, P, S, Si, F, Cl, Br or I. As above, there is an extra breakdown by the numbers of fluorine present overall for the sections with counts above 100.

PFAS and Fluorinated Compound Collections

The "*PFAS and Fluorinated Compound Collections*" section of the PubChem PFAS tree contains various lists gathered across PubChem content (see Figure 9). The mapping files to construct this are kept on the eci/pubchem repository on GitLab. Currently, the content displayed in Figure 9 comes from:

- All PFAS lists from the CompTox Chemicals Dashboard [8] via the EPA DSSTox Tree in PubChem;
- All PFAS lists from the NORMAN Suspect List Exchange (NORMAN-SLE) via the NORMAN-SLE Tree in PubChem;
- The CORE and Patent PFAS lists from OntoChem [9];
- Other collections from within PubChem Classification Trees, including collections from Cameo, ChEBI and MeSH;
- The NIST PFAS Suspect list list provided by Benjamin Place [10].

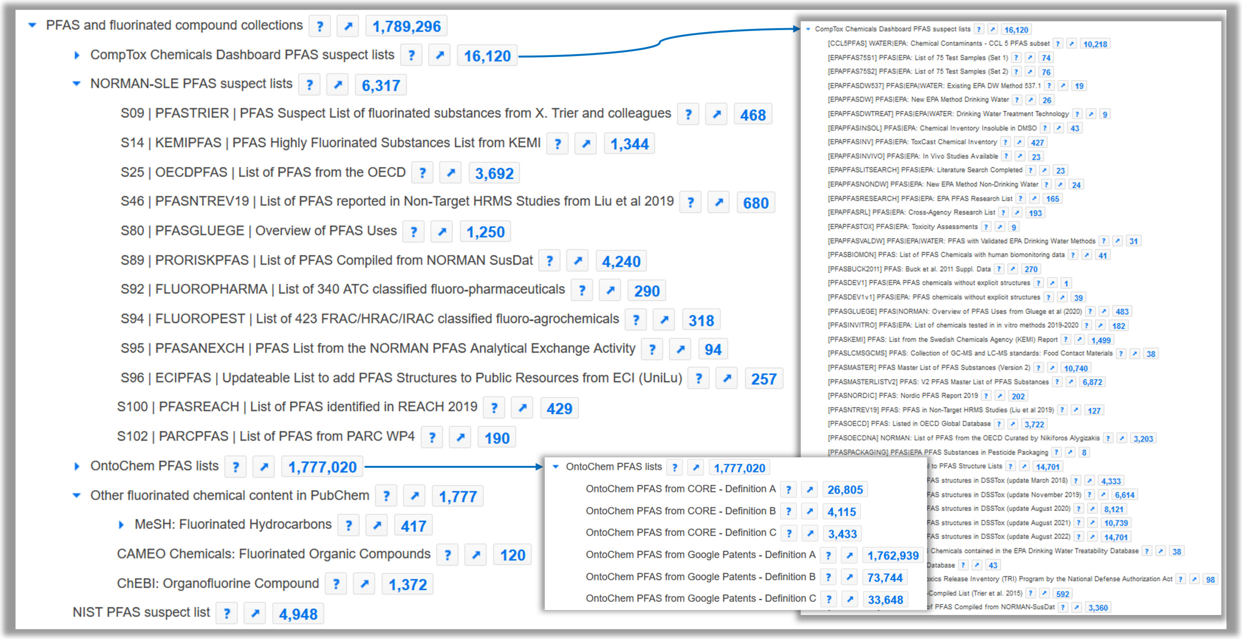

Figure 9: *The "PFAS and Fluorinated Compound Collections" node, with all major collections shown (CompTox and OntoChem as insets). Numbers and content listing from 11 Sept. 2023.*

Additional community-based PFAS can also be added to this section. Ideas and suggestions for new lists are welcome and will be added if feasible and possible. Please email suggestions or ideas to pubchem-help@ncbi.nlm.nih.gov or Emma Schymanski.

Regulatory PFAS Collections

Several regulatory PFAS collections from a variety of regulatory documents are currently included in the PubChem PFAS Tree as listed below, shown in Figures 10 & 11 and documented (as slides) in [11]. Please note that this section of the PubChem PFAS Tree is currently in active development with the community. Please email suggestions or ideas to pubchem-help@ncbi.nlm.nih.gov or Emma Schymanski directly. The use of SMARTS is explained in the tooltips.

The regulatory PFAS collections currently include:

- Long-chain perfluorocarboxylic acids (LC-PFCAs) and related substances
  - C9-C21 LC-PFCAs as nominated for the Stockholm Convention
- Perfluorohexane sulfonic acid (PFHxS) and related substances
  - PFHxS and related compounds as defined in Annex A of the Stockholm Convention
  - PFHxS (linear or branched) plus its salts and related substances according to EU REACH (draft definition)
  - Difference between Annex A and EU REACH definitions
- Perfluorooctanoic acid (PFOA) and related substances
  - PFOA and related compounds as defined in Annex A of the Stockholm Convention
- PFOA and related substances - exclusions
- Perfluorooctane sulfonic acid (PFOS) and related substances
  - PFOS, PFOSF and related substances as defined in Annex B of the Stockholm Convention

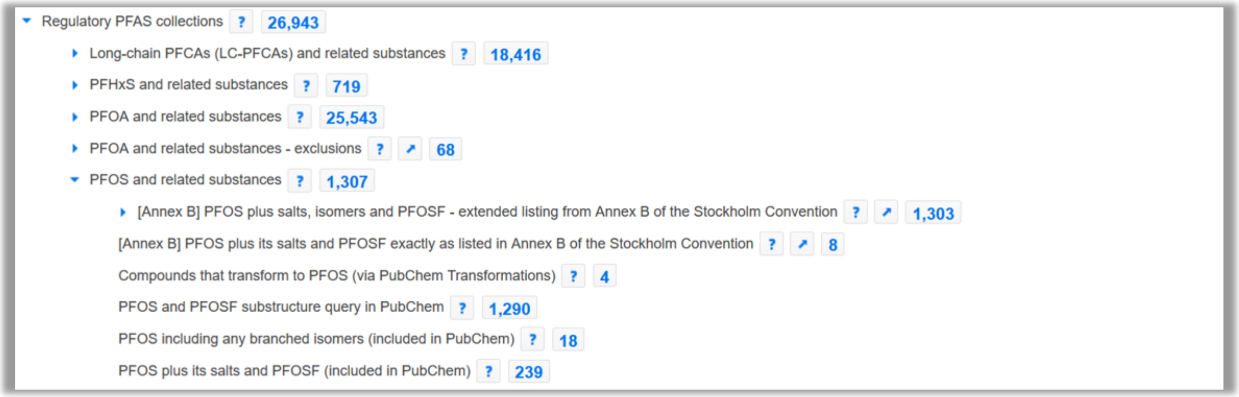

Figure 10: *The "Regulatory PFAS collections" part of the PubChem PFAS Tree, showing the major classes covered and a more detailed breakdown for PFOS (11 Sept. 2023).*

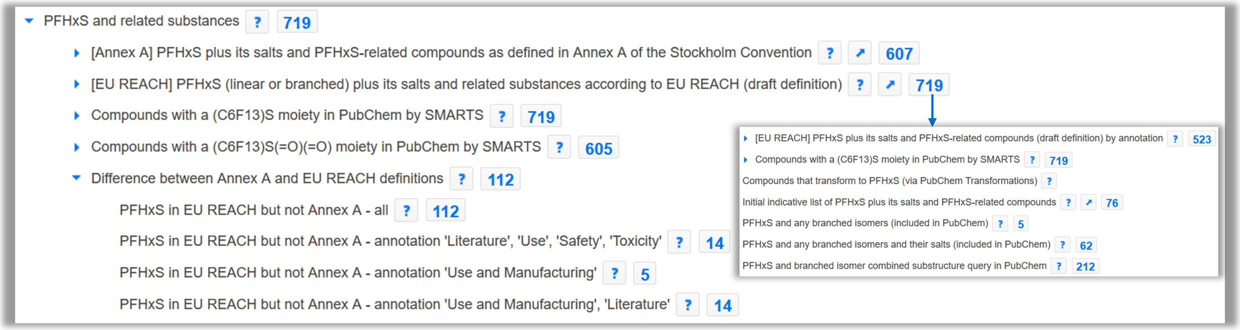

Figure 11: *The "Regulatory PFAS collections" part of the PubChem PFAS Tree, showing a partial breakdown for the PFHxS subsection, including annotation breakdown (11 Sept. 2023).*

As shown in the figure above, the regulatory collections also include detailed breakdowns of the contents according to annotation information present in the download files (described further in Section Download via PubChem Search). A section including recent CIDs is also included in the major regulatory definitions, allowing users to find relevant (by annotation) and recent (by date) entries. Categories follow the major headings of the PubChem Table of Contents and are patents, literature, use, safety and toxicity information.

PFAS Breakdowns by Chemistry

The PFAS breakdowns by chemisty section is an expansion of the OECD PFAS definition that also includes salts and mixtures, not just neutral compounds. This section contains four major breakdowns, by *composition* (neutral vs. salt/mixture), by *functional groups*, by *connectivity degree* (PFAS part connected to one or more non-PFAS parts) and by *PFAS part formulas* (i.e., length of the PFAS), as shown in Figures 12 and 13.

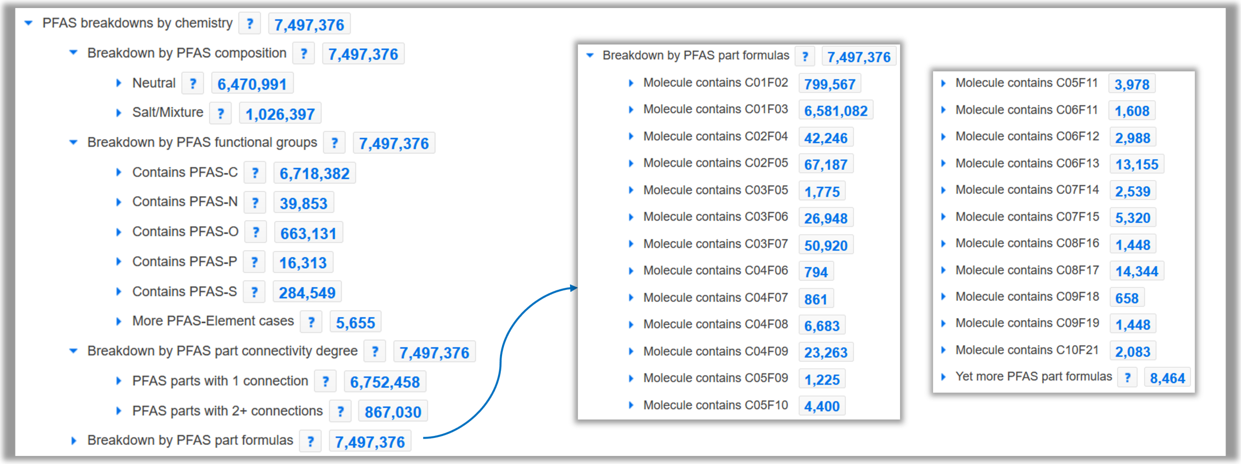

Figure 12: *The "PFAS breakdowns by chemistry" part of the PubChem PFAS Tree, showing the four major nodes and the first sublayer of each. Numbers from 11 Sept. 2023.*

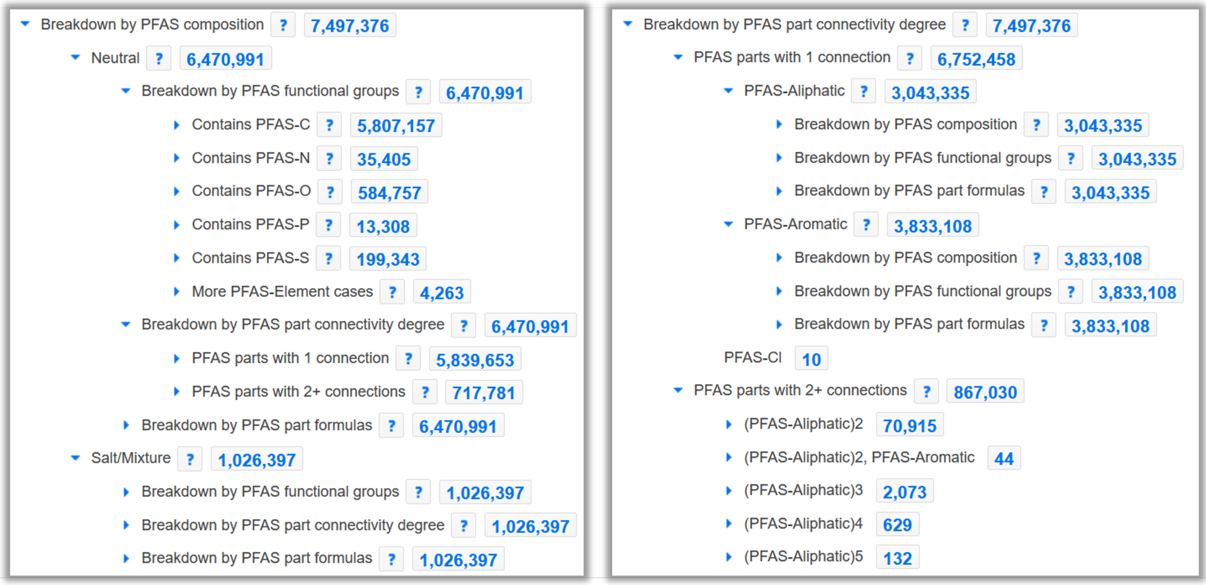

Figure 13: *Substructure of the "Breakdown by PFAS composition" and "Breakdown by PFAS part connectivity degree" sections, showing how each section can be broken down by the other categories. Numbers from 11 Sept. 2023.*

This section can be used to explore many functional properties about PFAS compounds, more examples will be shown in the following sections.

Exploring the PubChem PFAS Tree

While the tree offers several possibilities for browsing and searching PFAS and other organofluorine content, there are more powerful search capabilities to empower this further, as explained in the next sections.

Download via PubChem Search

Perhaps the most intuitive interaction is directly through clicking on the numbers besides each node (see Figure 14). This sends a query directly to the PubChem Search interface and displays the entire node contents, as shown in Figure 14. This query follows "*OECD PFAS definition*" > "*Molecule contains PFAS parts larger than CF_2/CF_3*" > "*Breakdown by isolated PFAS part count*" > "*Contains 01 isolated PFAS part*" > "*Count of molecules 10001-100000*" > "*Contains 01xC04F09-linear*" and returns 11,957 CIDs (20 June 2023) containing only one single linear C_4F_9 PFAS part. This query can then be downloaded or saved (Figure 14, insets), or sent to Entrez for advanced querying (see section on Entrez). Note that clicking on the "?" beside a node (where present) will open a tool tip explaining the node contents (Figure 14, bottom left).

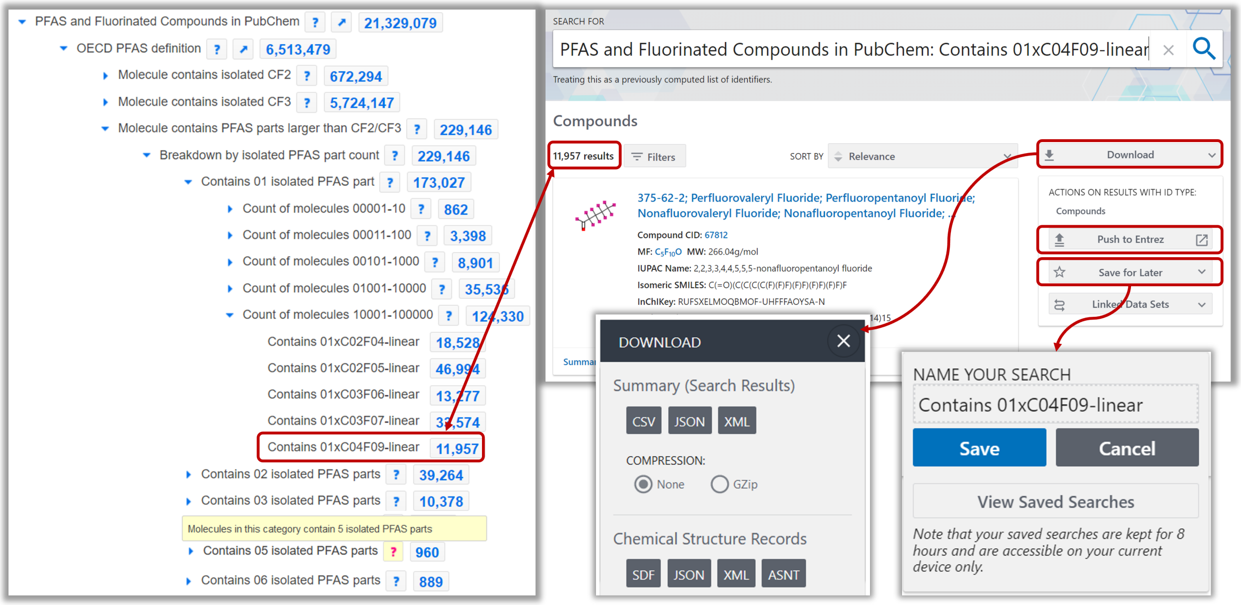

Figure 14: *Querying node contents in PubChem Search. When clicking on the blue numbers (left), a search window will open in a new tab (right, main image). This collection can be browsed, downloaded or saved (see insets) or sent to Entrez (see next section). Clicking on the "?" sign next to a node name will open a tool tip (left panel, bottom, see yellow blurb). Figure updated 20 June 2023.*

The download file contains a number of fields of interest, highlighted in Figure 15, including: PubChem compound identifier (CID), names and synonyms, several properties (*e.g.* XlogP, molecular formula, masses), structural information (SMILES, InChI, InChIKey), patent and literature counts as well as several metadata entries. These metadata entries contain valuable information about the evidence contributing to the presence of that structure in PubChem (*e.g.,* contribution source(s) and date, annotation information). Relevant fields are explained in Table 2 and shown in Figure 15.

Note that the categories visible in the "*annothits*" column align with the individual sections in PubChem records and can also be viewed in the PubChem Table of Contents (TOC) Tree. For any entry with annotation, the information available can be viewed for that individual CID. For example, the annotation information for CID 67814 in Figure 15 can be viewed for the following sections (selected examples): Classification, Names and Identifiers, Patents, Safety and Hazards, Use and Manufacturing.

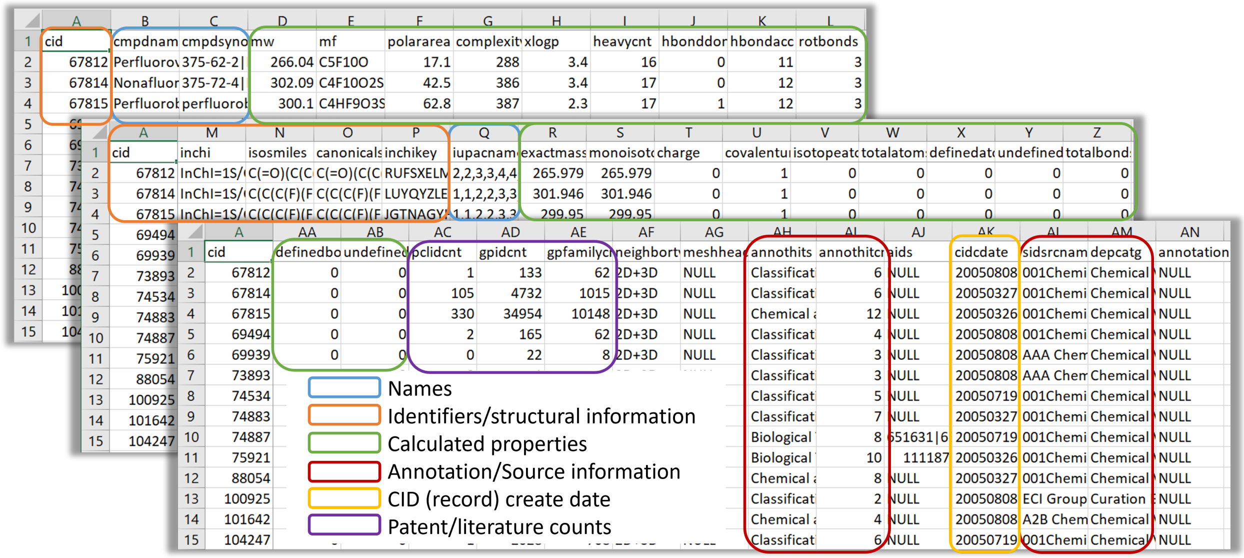

Figure 15: *PubChem Download file. Top left: PubChem Compound Identifier (CID), names, properties. Middle: structural information, names, more properties. Bottom: more properties, patent and literature counts, annotation content, CID dates. Downloaded from the query shown in Figure 14 on 20 June 2023.*

Table 2: *Relevant metadata files in the PubChem Download files.*

Header	Description	Type
annothits	Annotation categories present for this CID	Text
annothitcount	Count of annotation categories for CID	Numeric
cidcdate	CID creation date	YYYYMMDD
depcatg	Deposition category, reveals what type of sources contributed information	Text
pclidcnt	Consolidated literature count	Numeric
gpidcnt	Patent count	Numeric
sidsrcname	Name of the data source(s) contributing substance (SID) information for given CID	Text

There are many records where the information has only been extracted from patents, or for which no annotation exists. Thus, the various metadata fields listed in Table 2 can help add a lot of context to the relevance of the entries for the particular question at hand. More advanced queries are possible to leverage this information even further, as explained in the next sections.

PubChem "Saved Searches"

The PubChem "Saved Searches" feature can be used to save and interact with different searches using the Boolean operators "AND", "OR" and "NOT". Any section of any classification browser can be sent to PubChem Search (or uploaded via the "Upload ID list" option on the landing page). For instance, the saved searches shown in Figure 16 can be used to find out how many Agrochemicals are OECD PFAS with data in MassBank.EU. The window shown in Figure 16 was created by saving the "PFAS breakdowns by chemistry" section of the PubChem PFAS Tree as "OECD PFAS (incl salt/mix)", then saving the "Agrochemical Information" section of the PubChem Compound TOC Tree as "Agrochemicals", then saving the "Information Sources > MassBank Europe" section of the PubChem Compound TOC Tree as "MassBank Europe", then using the "AND" functionality at the top to build the respective queries.

These results can be viewed again in the PubChem Search interface (shown in Figure 14) and sent to Entrez (explained in next section) to see *e.g.*, the breakdown of Agrochemicals according to various categories of the PubChem PFAS Tree as shown to the right of Figure 16.

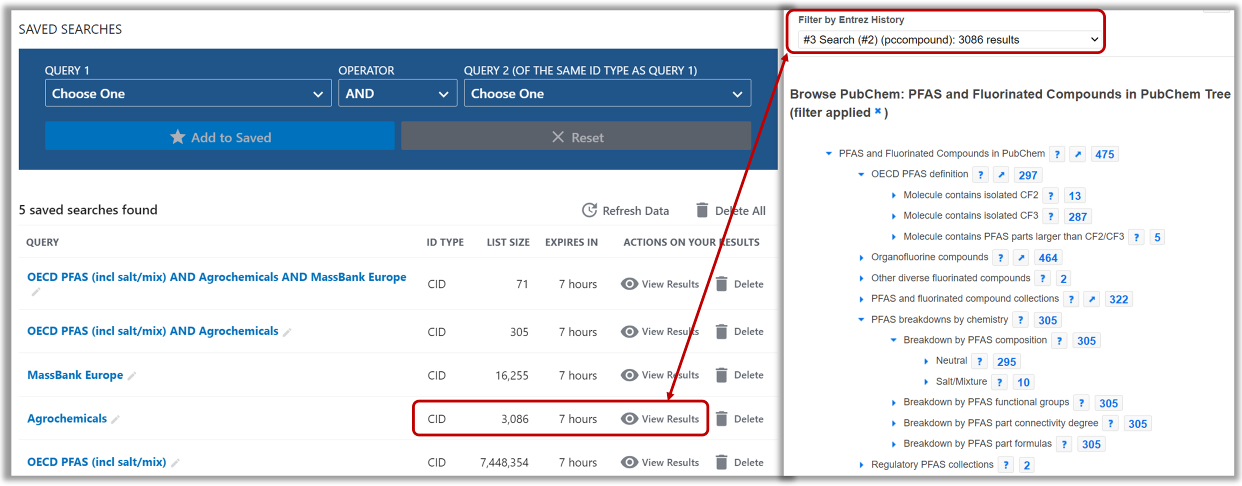

Figure 16: *Left: The Saved Searches interface in PubChem, with query builder at the top. Right: Viewing the results will open a PubChem Search window where the results can be sent to Entrez (see Figure 14) and then selected from a dropdown menu and browsed in the PubChem PFAS Tree (a refresh may be necessary). Created 21 June 2023.*

Interactions via Entrez

It is possible to build more extensive queries via the Entrez interface, which is accessible through the button below the download button (see Figure 14) or by clicking the "Use Entrez" option on the PubChem landing page. More documentation on Entrez is given here. It is also possible to send queries to Entrez via the PubChem Identifier Exchange Service (ID Exchange), as shown in Figure 17.

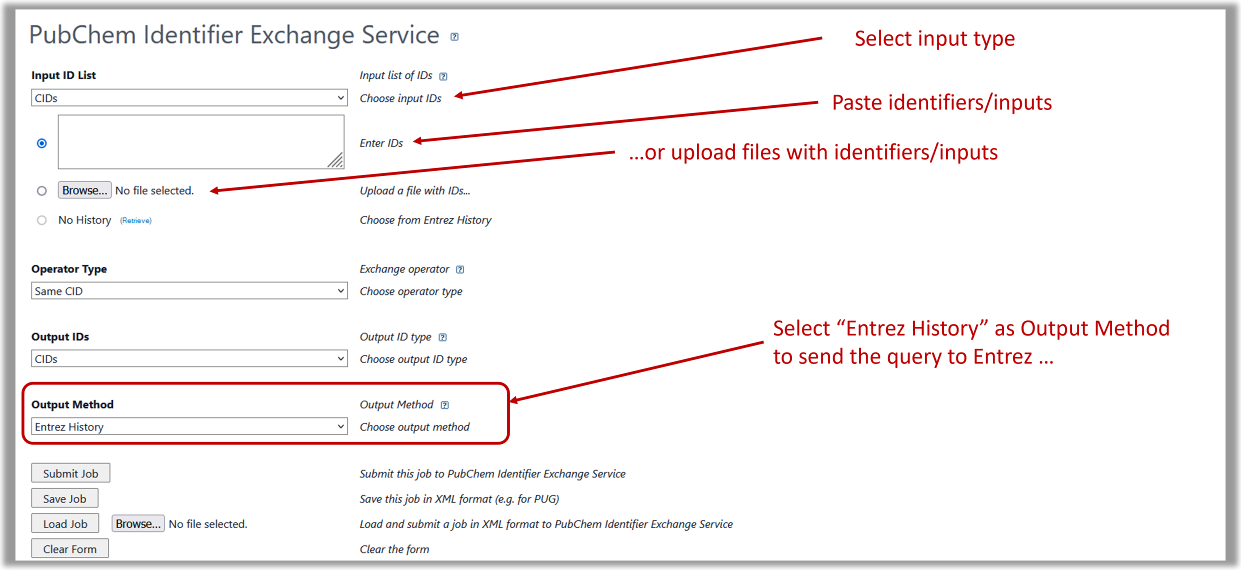

Figure 17: *Sending queries to Entrez via the PubChem ID Exchange.*

This rest of this section steps through a few interactive examples.

Example 1: Find all PFAS containing one linear C_4F_9 part with use information: To find all molecules from the query in Figure 14 that also have use information in PubChem, the first step is to send the 11,957 CIDs from the query above to Entrez via the "Push to Entrez" option (Figure 14, second box encircled in red on the right). This opens a new page in the Entrez interface (not shown). Next, go to the "Use and Manufacturing" section of the PubChem TOC Tree, send this to PubChem Search via the numbers next to the node (Figure 18, red circle on left), and push to Entrez (Figure 19, top right). By selecting the "Advanced" option under the search bar (Figure 18, top), the Advanced Search builder is opened and further queries can be built. By selecting "#5 AND #9", only the 437 chemicals with a single C_4F_9 linear PFAS part (query #5) that also have use and manufacturing information in PubChem (query #9) are returned.

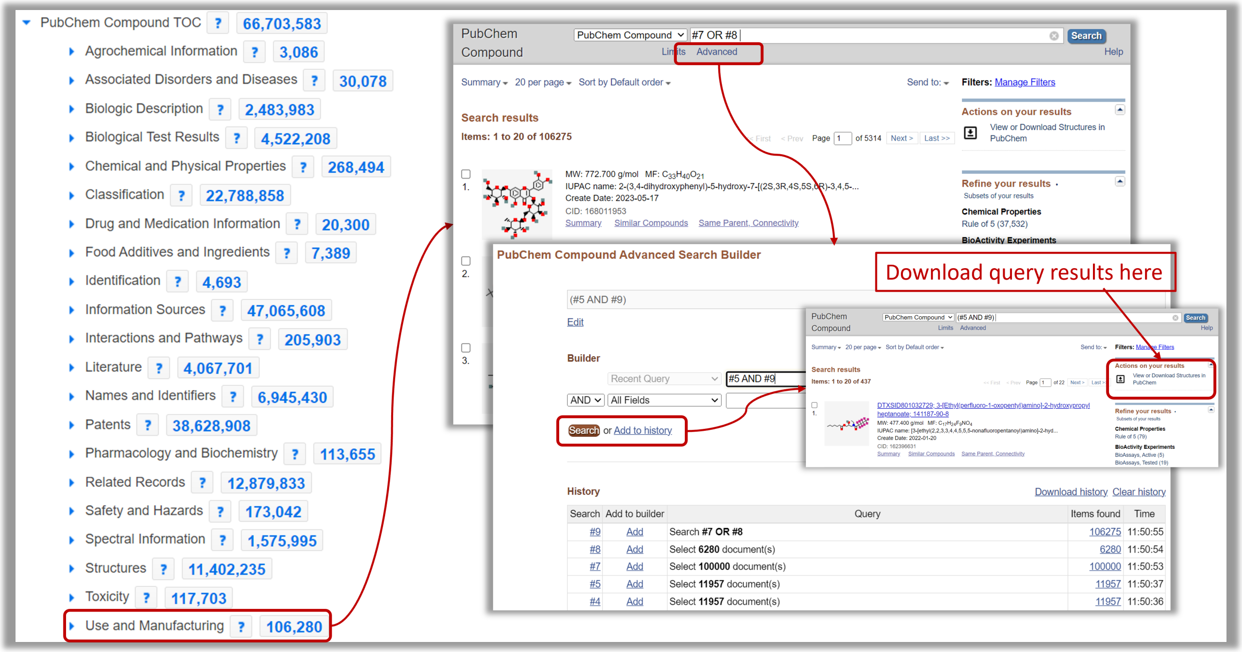

Figure 18: *Advanced search via Entrez. Left: PubChem TOC Tree. Top right: the Use and Manufacturing query in Entrez. Bottom right: the Advanced Search builder in Entrez, where query #5 (one C_4F_9 part only) AND #9 (Use information) is built. This is then sent again to search via Entrez (middle right) and the 437 C_4F_9 compounds with use information can be browsed or downloaded via the "View or Download Structures in PubChem" option. Queries run on 21 June 2023.*

Example 2: Browse all OECD PFAS with mass spectrometry information: The Entrez functionality can be used to find out which PFAS or organofluorine compounds have mass spectrometry information available in PubChem (or in resources integrated within PubChem). The tree contents can be subset according to other available information, as shown in Figure 19. First, go to the "Mass Spectrometry" section of the PubChem TOC Tree, under the "Spectral Information" heading, and send this query to Entrez (see Figure 19 left and top right). Then, go back to the PubChem PFAS Tree and ***refresh*** the contents. A new dropdown menu will appear (if not already present) called "Filter by Entrez History" (Figure 19, bottom right). By selecting the chosen query in this dropdown menu, the tree will then be subset by the contents within that query, such that only CIDs that are in the tree *and* in the query will show (in Figure 19, ~56K not 21M CIDs). The same holds for any advanced query, so it would be possible to *e.g.* do a subset of only mass spectra that occur in MassBank EU or NIST by additionally adding the relevant "Information Sources" (from the PubChem TOC Tree) to the Entrez query. Since large queries such as the "Mass Spectrometry" category, or advanced AND/OR combinations can end up quite complicated, noting the query number (#XXX) and the number of compounds in the result can be helpful. Alternatively, use the Saved Searches functionality to name the searches before sending them to Entrez (Figure 16).

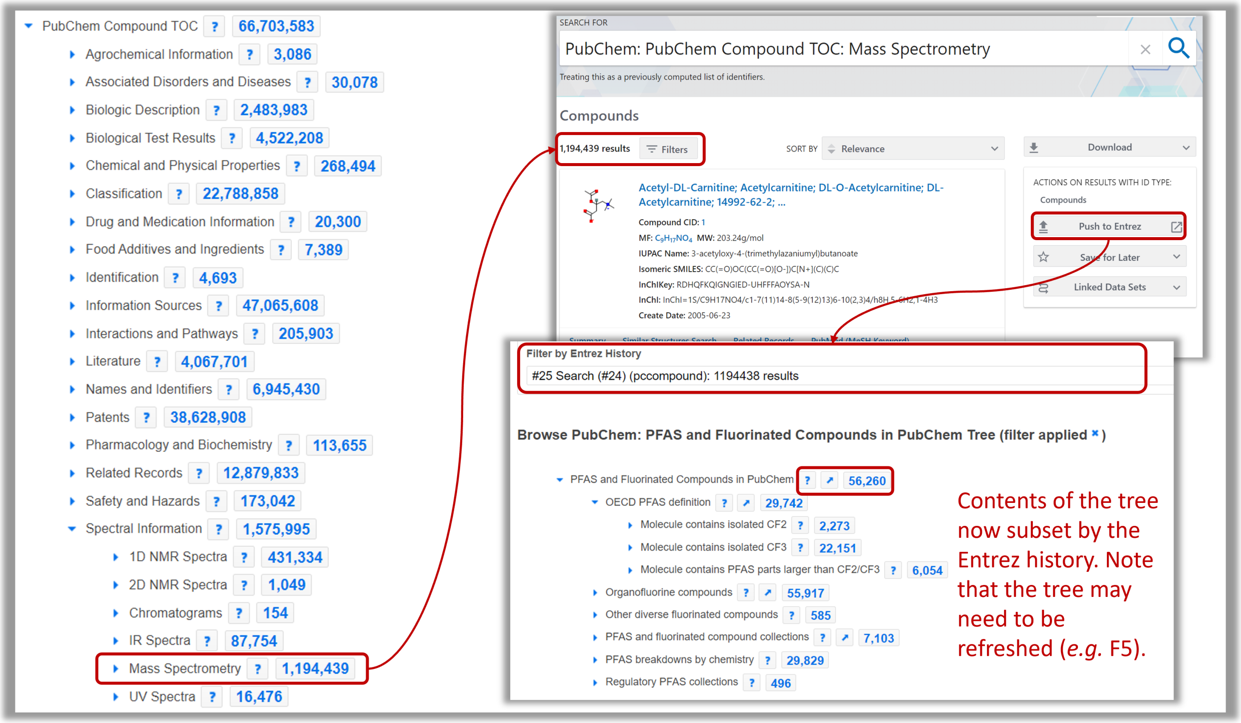

Figure 19: *Subsetting Tree Contents via Entrez. Left: PubChem TOC Tree, "Mass Spectrometry" subsection. Top right: the "Mass Spectrometry" query in PubChem Search (to be sent to Entrez). Bottom right: the PubChem PFAS Tree subset by Mass Spectrometry, now only displaying CIDs where mass spectrometry information is available in PubChem. Queries run on 21 June 2023.*

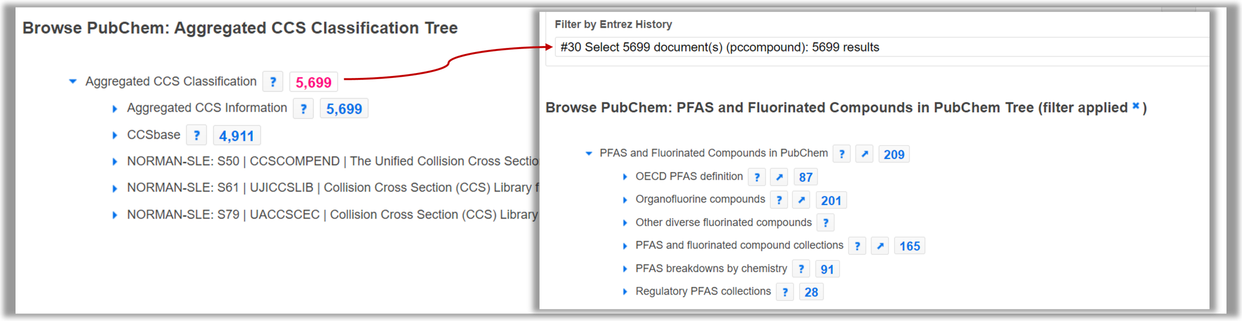

Figure 20: *Subsetting Tree Contents via Entrez. Left: Aggregated CCS Classification Tree. Right: the PubChem PFAS Tree subset by CCS values, now only displaying CIDs where collision cross section information is available in PubChem. Queries run on 21 June 2023.*

Example 3: Browse all PFAS with CCS information: Figure 20 (previous page) shows how to explore the PFAS with collision cross section (CCS) values using the Aggregated CCS classification.

Extra Details

This documentation is primarily aimed at describing the features of the PubChem PFAS Tree. This section includes some additional technical details, which will be expanded as further questions arise.

Programmatic Interactions via PUG REST

It is possible to interact with the PubChem PFAS Tree programmatically. For more extensive details on PUG REST and other programmatic access than contained below, please see the following locations in the PubChem documentation:

- https://pubchem.ncbi.nlm.nih.gov/docs/programmatic-access
- https://pubchem.ncbi.nlm.nih.gov/docs/pug-rest
- https://pubchem.ncbi.nlm.nih.gov/docs/pug-rest-tutorial
- https://pubchem.ncbi.nlm.nih.gov/docs/pug-rest#section=Classification-Nodes

Example code describing how to interact with the PubChem PFAS Tree is provided in a separate document, available as PFAS_Tree_in_R.pdf or PFAS_Tree_in_R.Rmd.

Areas of Development

There are currently several areas of active development, including:

- Handling of ethers and other connecting atoms;
- Adding salts/mixtures into the organofluorine and other fluorinated content sections
- Handling of polymer and poorly defined entries.

Polymers/Poorly defined entries: Since the entire PubChem PFAS Tree is constructed on CIDs (*i.e.*, compounds), substance entries (denoted by substance identifiers, SID) are not included. Thus, undefined or poorly defined entities and polymers are not included (such as the example in Figure 21). More information about the difference between compound and substances on PubChem is available here.

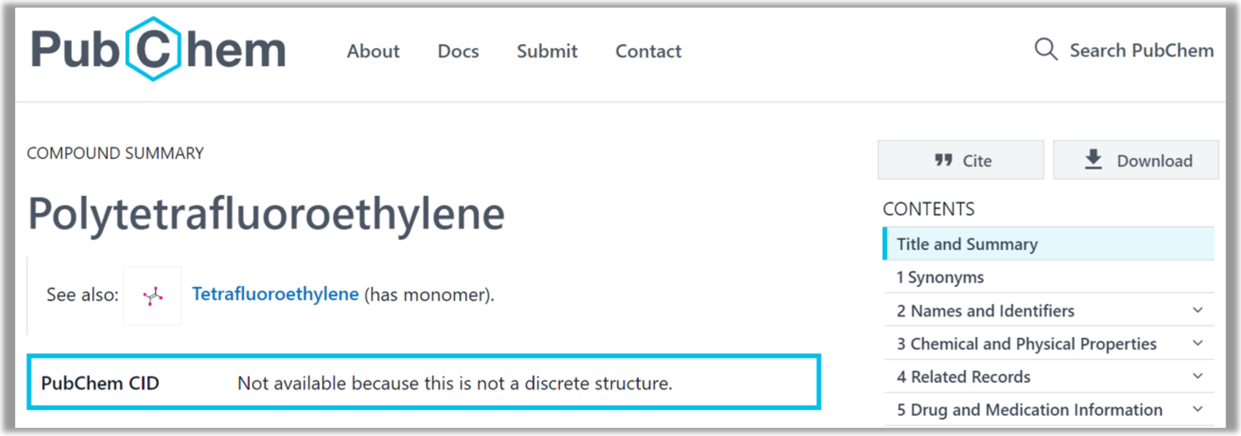

Figure 21: *An example of a polymer not yet included in the PFAS Tree - Teflon.*

PFAS Test set

A test set of PFAS and non-PFAS from the OECD Report [4] has been compiled to check the performance of the PubChem PFAS Tree. The test set (XLSX) can be downloaded here. Other formats can be made available if requested (and if reasonably possible).

Downloading large files

Attempting to download nodes containing millions of entries can result in download files that exceed Microsoft Office size limits. Adjusting this example download URL can be used to select columns and row numbers, to navigate around the limits. Please note that the cache key will have to be replaced by an active download query cache key for this URL to work.

Contact Details

User feedback is extremely valuable to help improve this tree further. Please reach out to either contact author (details on first page, or email Evan and Emma directly) with feedback and comments! Suggestions for PFAS or fluorinated compound collections to include in the "*PFAS and Fluorinated Compound Collections*" section of the PubChem PFAS Tree can be sent to pubchem-help@ncbi.nlm.nih.gov or Emma Schymanski directly.

For general questions about PubChem and the functionality described here, please reach out to the PubChem Help mailing list for further support.

Statements

Author Contributions

ELS: Conceptualization (equal), data curation, methodology, software, validation, writing - original draft preparation, writing - review and editing. PC: Validation (supporting). TK: Software. PAT: Data curation, methodology, software. JZ: Data curation, methodology, software. EEB: Conceptualization (equal), data curation, methodology, software (lead), validation, writing - original draft preparation, writing - review and editing.

Acknowledgements

We would like to acknowledge discussions with Zhanyun Wang (EMPA, CH), Hans Peter Arp (NGI, NO), Ian Cousins, Luc Miaz, Jon Martin (ACES, SE), as well as other project members of ZeroPM. We acknowledge many valuable discussions with Andreas Buser (FOEN, Switzerland) during development of the Regulatory collections [11]. We would also like to acknowledge discussions and contributions from various members of the ECI and PubChem teams that were not directly involved in these efforts but have contributed indirectly through our many other collaborative efforts!

References

1. Kim S, Chen J, Cheng T, et al (2022) PubChem 2023 update. Nucleic Acids Research gkac956. https://doi.org/10.1093/nar/gkac956
2. Schymanski E, Bolton E (2023) ZeroPM Webinar: Are there really 6 million PFAS in PubChem? Zenodo. https://doi.org/10.5281/zenodo.7756622
3. Schymanski E, Zhang J, Thiessen P, et al (2023) Per- and polyfluoroalkyl substances (PFAS) in PubChem: 7 million and growing. ChemRxiv. https://doi.org/10.26434/chemrxiv-2023-j823z
4. OECD (2021) Reconciling Terminology of the Universe of Per- and Polyfluoroalkyl Substances: Recommendations and Practical Guidance. https://www.oecd.org/chemicalsafety/portal-perfluorinated-chemicals/terminology-per-and-polyfluoroalkyl-substances.pdf. Accessed 14 Nov 2021
5. Mayfield J (2022) CDK Depict Web Interface. http://simolecule.com/cdkdepict/depict.html. Accessed 24 Mar 2022
6. Ruttkies C, Schymanski EL, Wolf S, et al (2016) MetFrag relaunched: Incorporating strategies beyond in silico fragmentation. Journal of Cheminformatics 8:3. https://doi.org/10.1186/s13321-016-0115-9
7. Schymanski E, Bolton E, Chirsir P, et al (2022) PubChem OECD PFAS Larger PFAS Parts file for MetFrag. Zenodo. https://doi.org/10.5281/zenodo.6385954

8. Williams AJ, Grulke CM, Edwards J, et al (2017) The CompTox Chemistry Dashboard: A community data resource for environmental chemistry. Journal of Cheminformatics 9:61. https://doi.org/10.1186/s13321-017-0247-6

9. Barnabas SJ, Böhme T, Boyer SK, et al (2022) Extraction of chemical structures from literature and patent documents using open access chemistry toolkits: A case study with PFAS. Digital Discovery 1:490–501. https://doi.org/10.1039/D2DD00019A

10. Place B (2021) Suspect List of Possible Per- and Polyfluoroalkyl Substances (PFAS). https://data.nist.gov/od/id/mds2-2387. Accessed 29 Sep 2022

11. Schymanski E, Bolton E (2022) How can the "PubChem PFAS Tree" Help Support the Regulation of PFAS? https://doi.org/10.5281/zenodo.7118551
